# Genome-Wide analysis of the AAAP gene family in moso bamboo (*Phyllostachys edulis*)

**DOI:** 10.1186/s12870-017-0980-z

**Published:** 2017-01-31

**Authors:** Huanlong Liu, Min Wu, Dongyue Zhu, Feng Pan, Yujiao Wang, Yue Wang, Yan Xiang

**Affiliations:** 1 Laboratory of Modern Biotechnology, School of Forestry and Landscape Architecture, Anhui Agricultural University, Hefei, 230036 China; 2 Key Laboratory of Crop Biology of Anhui Province, School of Life Sciences, Anhui Agricultural University, Hefei, 230036 China; 3 National Engineering Laboratory of Crop Stress Resistance Breeding, School of Life Sciences, Anhui Agricultural University, Hefei, 230036 China

**Keywords:** Moso bamboo, Amino acid/auxin permease, Phylogenetic analysis, Conversed motif, Expression patterns, qRT-PCR

## Abstract

**Background:**

Members of the amino acid/auxin permease (AAAP) gene family play indispensable roles in various plant metabolism and biosynthesis processes. Comprehensive analysis of AAAP genes has been conducted in Arabidopsis, rice, maize and poplar, but has not been reported from moso bamboo. Phylogenetics, evolutionary patterns and further expression profiles analysis of the AAAP gene family in moso bamboo (*Phyllostachys edulis*) will increase our understanding of this important gene family.

**Results:**

In this current study, we conducted phylogenetic, gene structure, promoter region, divergence time, expression patterns and qRT-PCR analysis of the 55 predicted AAAP genes in moso bamboo based on the availability of the moso bamboo genome sequence. We identified 55 putative AAAP (*PeAAAP1-55*) genes, which were divided into eight distinct subfamilies based on comparative phylogenetic analysis using 184 full-length protein sequences, including 55 sequences from moso bamboo, 58 sequences from rice and 71 sequences from maize. Analysis of evolutionary patterns and divergence showed that the PeAAAP genes have undergone a extensive duplication event approximately 12 million years ago (MYA) and that the split between AAAP family genes in moso bamboo and rice occurred approximately 27 MYA. The microarray analysis suggested that some genes play considerable roles in moso bamboo growth and development. We investigated the expression levels of the 16 AAP subfamily genes under abiotic stress (drought, salt and cold) by qRT-PCR to explore the potential contributions to stress response of individual *PeAAAP* genes in moso bamboo.

**Conclusions:**

The results of this study suggest that *PeAAAP* genes play crucial roles in moso bamboo growth and development, especially in response to abiotic stress conditions. Our comprehensive, systematic study of the AAAPs gene family in moso bamboo will facilitate further analysis of the functions and evolution of *AAAP* genes in plants.

**Electronic supplementary material:**

The online version of this article (doi:10.1186/s12870-017-0980-z) contains supplementary material, which is available to authorized users.

## Background

Amino acids are important organic substances that serve as an indispensable source of organic nitrogen for growth and development, playing vital roles in the metabolism, structure and biosynthesis of various compounds in eukaryotic organisms [1, 2]. In plants, amino acids are important components of nucleotides, chlorophyll, phytohormones and secondary metabolites [2]. Amino acids are transported between different organs through both xylem and phloem, requiring the activity of amino acid transporters (AATs) in the plasma membrane [3]. The first plant amino acid transporter was found in Arabidopsis 23 years ago, namely *AtAAP1/NAT2* [4, 5]. Amino acid/auxin permease (AAAP) proteins are found in almost all eukaryotic organisms, belonging to the AAT family [1, 6, 7]. These proteins contribute to the responses to biotic and abiotic stresses and long distance amino acid transport, and they mediate the transport of amino acids across the cellular membrane [8–10]. In addition, previous reports showed that some members of amino acid transporters were located within the tonoplast, which were devoted to transport amino acids between vacuole and cytoplasm, and regulated the storage of amino acids in vacuole [11–14].

To date, the AAAP family is one of the largest families of AATs [1, 6, 7], comprising eight subfamilies, namely ProTs [15], GATs [16], LHTs [17], AAPs [1, 18], ANTs [12] and ATL subfamilies (ATLa and ATLb) [19]. And all *AAAP* genes have a specific domain, PF01490 (Aa_trans).

To date, some functions of AAAP proteins have been studied in model plants such as Arabidopsis [20], poplar [21], maize [22] and rice [23]. *AtAAP3* appears to be involved in amino acid uptake from the phloem and soil [24]. A recent study showed that *AtAAP5* plays a role in amino acid uptake by the root [1]. *AtAAP6* is expressed in roots, sink leaves, cauline leaves and xylem parenchyma, suggesting that it functions in amino acid uptake from the xylem [25]. In addition, *AtAAP8* might play a crucial role in amino acid transport during fruit development [1, 26]. In rice, 18 genes in the AAP subfamily have been identified [23], three of which (*OsAAP1*, *OsAAP7* and *OsAAP16*) encode general AAAP proteins, whereas *OsAAP3* does not [27]. *OsAAP3* transports the basic amino acids lysine and arginine and has distinct substrate specificity compared with other rice or *Arabidopsis* AAPs [27]. *OsAAP6* is contribute to enhance root absorption and affect the distribution of various amino acids in early stages of seed development [28].

Bamboo, one of the most important non-timber forest products worldwide, comprises over 70 genera and 1,200 species [29]. A majority of these species are distributed in the subtropical regions of China, especially regions south of the Yangtze River. Moso bamboo is an important species in China with the highest value in several areas among all bamboos, being used to produce timber, paper, artwork and food (young shoots) [30]. However, moso bamboo faces many types of environmental conditions during growth and development, such as high or low temperatures, salt concentrations and soil moisture levels, which limit its distribution and quality. A previous study showed that functional and regulatory proteins contribute to abiotic stress resistance in plant [31], and AAAP proteins are the fundamental functional proteins. Therefore, in the current study, we investigated AAAP proteins in moso bamboo to identify proteins that function in stress resistance. To date, bioinformatic analysis in model plants has greatly increased our understanding of *AAAP* genes. In addition, the draft genome sequence of moso bamboo was completed in 2013 [29], providing a great bioinformatics foundation to perform a comprehensive genome survey of the AAAP family in moso bamboo.

## Methods

### Identification of moso bamboo AAAP genes

The conserved AAAP domains (PF01490) of rice AAAP protein sequences were originally applied as seed sequences to search the NCGR database (www.ncgr.ac.cn/bamboo) [29]. Redundant sequences were removed manually based on the results of Cluster W 2.11 alignment [32], and each candidate sequence was confirmed using the Pfam (http://pfam.xfam.org/) [33, 34] and SMART (http://smart.embl-heidelberg.de/) databases [35]. The number of amino acids, CDS lengths and physicochemical parameters of *AAAP* genes were obtained from Bamboo GDB (http://www.bamboogdb.org). Comparing coding sequence and the corresponding genomic DNA sequences of *AAAP* genes, we obtained their exon/intron structures from GSDS. The TMHMM Server version 2.0 (http://www.cbs.dtu.dk/services/TMHMM/) was used to predict the putative TM (transmembrane) regions of each PeAAAP protein with default settings.

### Phylogenetic and conserved motif analyses

Multiple sequence alignment was performed using ClustalX 2.11 software [36], and a phylogenetic tree was constructed based on the alignment with the N-J method using MEGA 6.0 software and bootstrap analysis of 1,000 replicates. The combined phylogenetic tree of OsAAAP, ZmAAAP and PeAAAP proteins was generated using the same method. The motifs of PeAAAP proteins were identified using the MEME tool (http://meme-suite.org/tools/meme) (parameter setting: maximum number of motifs, 20; maximum width, 50.).

### Calculation of Ka/Ks values

Pairwise alignment of *AAAP* genes encoding sequences of the orthologous and paralogous pairs was first performed using ClustalX 2.11 software and the results of alignment were subsequently further analyzed using the MEGA 6.0, and then the synonymous substitution rate (Ks) and nonsynonymous substitution rate (Ka) were computed using DnaSP 5 software [37, 38]. The divergence time (T) was calculated using the formula T = Ks/2λ (λ = 6.5 × 10^−9^) [29, 39]. The following parameters were used to perform sliding window analysis of the Ka/Ks ratios of all homologous gene pairs: window size, 150 bp; step size, 9 bp.

### Putative promoter region analysis

The 2,000-bp upstream sequences of the genetic sequences were identified as putative promoter regions, which contains various *cis-*regulatory elements identified using the PLACE website (http://www.dna.affrc.go.jp/PLACE/) [40].

### Plant material and growth conditions

Eight-week-old seedlings were grown in artificial growth chamber with a constant photoperiod (14 h light/8 h darkness) and temperatures average around 22 °C. Moso bamboo seeds for breeding seedlings were collected in the Tianmu Mountain National Nature Reserve in Zhejiang Province, China. In addition, the permission of seeds collection for the experiments was obtained from Prof. Dingqing Tang of School of Forestry and Bio-technology, Zhejiang A & F University. And the identification of these seeds was also performed by Prof. Dingqing Tang. The seedlings were treated with 20% PEG-6000, 200 mM NaCl and 4 °C to induce drought stress, salt stress and cold stress, respectively. In order to obtain reliable experimental data and reduce experimental error, for each sample, we executed three repeated trials for the same stimulation and carried out three biological replicates for expression analysis. For each induction treatment, we collected samples at six time points (0, 1, 3, 6, 12 and 24 h) and immediately stored at – 80 °C freezer for RNA extraction. In addition, untreated plant materials (0 h) were used as the control group.

### Expression profile analysis

To study gene expression levels of *PeAAAP* genes in different tissues or development stages. The expression profile for each gene was obtained from Short Read Archive (SRA) database of NCBI. And then the raw RNA-seq reads of BioProject ERP001341 were trimmed to remove low quality base-calls (Q < 20) and adaptor sequences with pipeline Fastq clean [41]. The paired clean reads were mapped to the *Phyllostachys heterocycla* reference genome using pipeline tophat2 with defaults parameters, and different expressed genes were detected by Cufflinks [42]. The heatmap of *PeAAAP* genes in seven different tissues and/or developmental stages (leaf, early panicle, advanced panicle, root, rhizome, 20-cm shoot and 50-cm shoot) was exhibited using the Heatmapper Plus tool [43].

### qRT-PCR analysis

To research the expression levels of *PeAAAP* genes, qRT-PCR analysis based on SYBR-green fluorescence was performed for each members of the AAP subfamily. Total RNA was extracted from the plant samples using RNA prep Pure Plant Kit (Tiangen) according to the manufacturer’s instructions, which was reverse transcribed into cDNA subsequently using a PrimeScript™ RT Reagent Kit (TaKaRa). Primer Express 3.0 was used to design the gene-specific primers of each *PeAAP* genes, and the tonoplast intrinsic protein 41 (TIP41) was used as an internal control [44]. The following program was used for qRT-PCR: 95 °C for 30 s; 40 cycles of 95 °C for 10 s, 55 °C for 15 s, 72 °C for 10 s.

## Results

### Identification of AAAP genes in moso bamboo

The AAAP candidate sequences from the moso bamboo genome were verified using the Pfam (http://pfam.xfam.org/) [33, 34] and SMART (http://smart.embl-heidelberg.de/) databases [35]. Fifty-five potential AAAP sequences were ultimately identified as AAAP genes, which were designated *PeAAAP01* to *PeAAAP55.* Detailed information about the 55 AAAP genes was obtained using the moso bamboo GDB server (http://www.bamboogdb.org), including the predicted lengths of CDSs, sizes of encoded proteins and physicochemical parameters; this information is shown in Table 1. The lengths of the CDSs range from 252 bp to 2,166 bp, with an average size of 1,281 bp. The identified AAAP genes in moso bamboo with an average size of 426 aa, which peak on 721 aa and have a minimum value at 83 aa. The predicted molecular weights of the 55 *PeAAAP* gene products range from 9.28 kDa (*PeAAAP3*) to 77.56 kDa (*PeAAAP10*), with a mean value of 46.48 kDa. The predicted PIs for the 55 *PeAAAP* gene products are below 11.0, with most values approximately 8.0 or 9.0. However, the PI of one gene product (*PeAAAP29*) is below 5.0, whereas one is greater than 10.0 (*PeAAAP37*).

**Table 1:** Detailed information about 55 predicted AAAP proteins in moso bamboo

Name	Gene ID	Location	CDS length(bp)	Protein			Exons
				Size (aa)	MW(Da)	pI	
PeAAAP1	PH01006117G0020	PH01006117:17405–21642	1002	333	36350.6	9.34	5
PeAAAP2	PH01239930G0010	PH01239930:32–575	420	139	15690.5	7.73	2
PeAAAP3	PH01003714G0090	PH01003714:68619–70863	252	83	9282.4	9.73	3
PeAAAP4	PH01003767G0090	PH01003767:54114–56085	468	155	16695.2	9.38	1
PeAAAP5	PH01002737G0060	PH01002737:82597–87807	1437	478	52505.7	8.56	6
PeAAAP6	PH01003320G0110	PH01003320:76173–81586	1341	446	49597.7	9.20	8
PeAAAP7	PH01004858G0010	PH01004858:2573–8555	1962	653	69948.0	9.48	6
PeAAAP8	PH01003226G0090	PH01003226:48722–54105	1440	479	53267.8	8.78	8
PeAAAP9	PH01005914G0030	PH01005914:9924–13146	1377	458	50863.3	8.97	4
PeAAAP10	PH01003455G0030	PH01003455:19503–26397	2166	721	77555.0	8.52	6
PeAAAP11	PH01006234G0010	PH01006234:49–3996	1593	530	57957.9	8.69	6
PeAAAP12	PH01001504G0170	PH01001504:91120–95101	1377	458	50064.7	6.12	5
PeAAAP13	PH01001524G0340	PH01001524:238692–245459	1452	483	53416.3	5.82	11
PeAAAP14	PH01001531G0320	PH01001531:229654–234370	1464	487	53243.7	8.75	6
PeAAAP15	PH01001798G0120	PH01001798:67305–71044	1359	452	49259.4	9.13	7
PeAAAP16	PH01001814G0060	PH01001814:44597–47519	1458	485	51720.2	6.37	1
PeAAAP17	PH01001871G0210	PH01001871:196645–199613	1389	462	50304.7	8.88	5
PeAAAP18	PH01001905G0370	PH01001905:265612–269395	1332	443	47937.0	8.83	3
PeAAAP19	PH01002263G0270	PH01002263:143302–145983	1371	456	50890.3	9.27	7
PeAAAP20	PH01002344G0230	PH01002344:136018–141071	1401	466	49740.4	9.02	6
PeAAAP21	PH01002444G0060	PH01002444:46449–49286	1044	347	38196.3	9.60	3
PeAAAP22	PH01001030G0470	PH01001030:274519–281336	1266	421	46576.7	8.42	11
PeAAAP23	PH01001101G0160	PH01001101:128998–132429	1296	431	47518.8	8.96	7
PeAAAP24	PH01001222G0340	PH01001222:244844–248327	1431	476	53315.3	8.93	6
PeAAAP25	PH01001336G0290	PH01001336:209093–210987	1446	481	51753.0	8.75	3
PeAAAP26	PH01001359G0300	PH01001359:218652–224100	1344	447	48641.7	8.84	6
PeAAAP27	PH01001376G0370	PH01001376:249070–252840	1374	457	49479.7	9.06	4
PeAAAP28	PH01001440G0160	PH01001440:109806–116337	1623	540	58764.0	6.03	12
PeAAAP29	PH01001440G0250	PH01001440:180216–187677	1323	440	48172.0	4.91	3
PeAAAP30	PH01000413G0700	PH01000413:450193–455278	1575	524	58205.4	8.90	7
PeAAAP31	PH01000455G0680	PH01000455:533312–535334	402	133	14970.3	9.97	5
PeAAAP32	PH01000563G0410	PH01000563:237821–239464	1302	433	46475.6	7.54	2
PeAAAP33	PH01000665G0180	PH01000665:118378–120284	1332	443	47788.8	8.73	3
PeAAAP34	PH01000192G0540	PH01000192:413803–416123	1305	434	46069.7	9.43	5
PeAAAP35	PH01000272G0710	PH01000272:434005–436104	1527	508	54788.7	9.18	5
PeAAAP36	PH01000282G1230	PH01000282:846946–852344	1536	511	55951.8	9.10	7
PeAAAP37	PH01000316G0910	PH01000316:582436–585525	1458	485	54547.3	10.30	8
PeAAAP38	PH01000317G0180	PH01000317:121776–124854	1455	484	52585.3	8.52	4
PeAAAP39	PH01000339G0320	PH01000339:204214–208734	1593	530	58732.2	8.85	7
PeAAAP40	PH01000351G0270	PH01000351:210267–213487	816	271	29564.6	9.03	3
PeAAAP41	PH01000366G0450	PH01000366:307920–311332	1347	448	48709.1	6.59	5
PeAAAP42	PH01000373G0290	PH01000373:201459–206733	1539	512	56757.8	8.78	8
PeAAAP43	PH01000000G4870	PH01000000:3182418–3184993	555	184	19761.7	6.54	1
PeAAAP44	PH01000004G2930	PH01000004:1884022–1886338	1026	341	37150.9	9.10	3
PeAAAP45	PH01000004G3230	PH01000004:2031818–2038930	1434	477	53155.2	8.27	7
PeAAAP46	PH01000005G2900	PH01000005:1803567–1805559	801	266	28398.3	8.86	3
PeAAAP47	PH01000005G2920	PH01000005:1811071–1813364	591	196	21654.4	7.68	5
PeAAAP48	PH01000009G3370	PH01000009:2240750–2242253	663	220	23653.9	9.25	1
PeAAAP49	PH01000041G0120	PH01000041:85713–89305	1377	458	50364.8	8.58	7
PeAAAP50	PH01000041G2190	PH01000041:1446042–1448569	1329	442	46803.6	8.14	3
PeAAAP51	PH01000061G0730	PH01000061:513980–516340	1461	486	51953.5	6.60	1
PeAAAP52	PH01000090G0310	PH01000090:175343–181904	1593	530	57324.9	8.97	8
PeAAAP53	PH01000090G0510	PH01000090:319750–326650	1482	493	54847.2	8.74	7
PeAAAP54	PH01000121G0220	PH01000121:148573–155266	1491	496	52929.2	9.18	7
PeAAAP55	PH01000122G1500	PH01000122:884662–888063	1266	421	44594.2	8.49	3

*CDS* coding sequence, *bp* base pair, aa amino acids, *MW* molecular weight, *pI* isoelectric point, Da Dalton

**Fig. 1:**
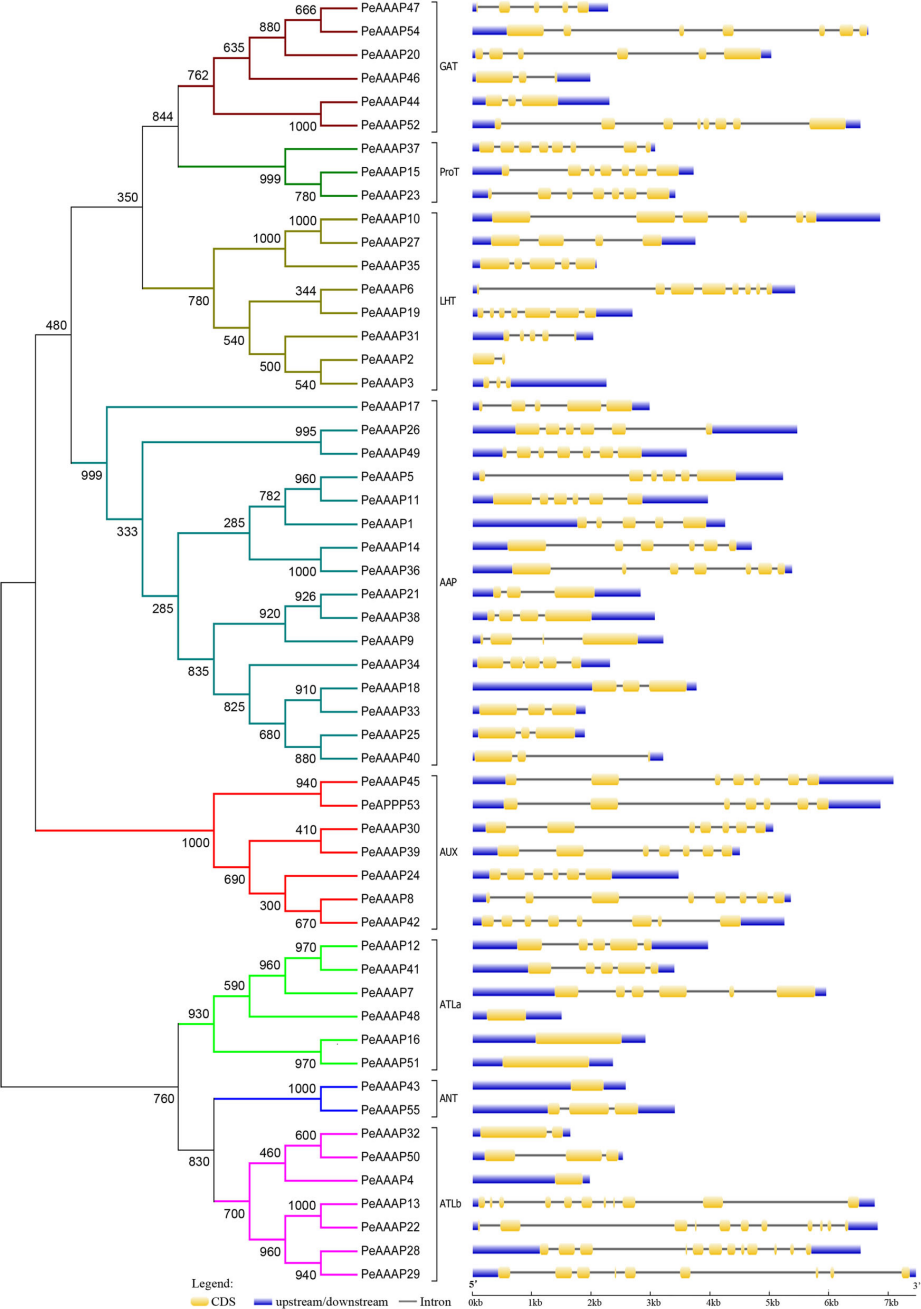
Phylogenetic relationship and gene structures of AAAP proteins in moso bamboo. *Left*: Phylogenetic tree of PeAAAPs constructed by neighbor-joining method based on the results of sequence alignment. Bootstrap values from 1000 replicates were indicated at each node. The proteins on the tree can be divided into eight distinct subfamilies and the branches of different subfamilies were marked by different colors. There was a special noted that subfamilies ATL were further divided into two groups (ATLa and ATLb). *Right*: Exons, introns and untranslated regions (UTRs) were indicated by yellow rectangles, gray lines and blue rectangles, respectively

The GSDS online tool was used to determine the exons/introns structure of each predicted *PeAAAP* gene (Fig. 1). These results show that five *PeAAAP* genes (*PeAAAP4*, *PeAAAP16*, *PeAAAP43*, *PeAAAP48* and *PeAAAP51*) exclude introns, while the remaining genes contain 1–11 introns, and *PeAAAP2* is no upstream and downstream (Fig. 1). The TMHMM Server v2.0 was used to predict the putative TM regions of *PeAAAP* genes, which numbers in most genes ranged from 8 to 13 (Additional file 1: Figure S1). Interestingly, we found that genes of the same subfamily have similar numbers of TMs, especially all members of AUXs contain 10 TMs. In AAP subfamily, the numbers of TMs was less than or equal to 10. However, a previous report, showing that *AtAAP1* had 11 TMs [45].

### Phylogenetic and conserved domain analysis of AAAP proteins in moso bamboo

**Fig. 2:**
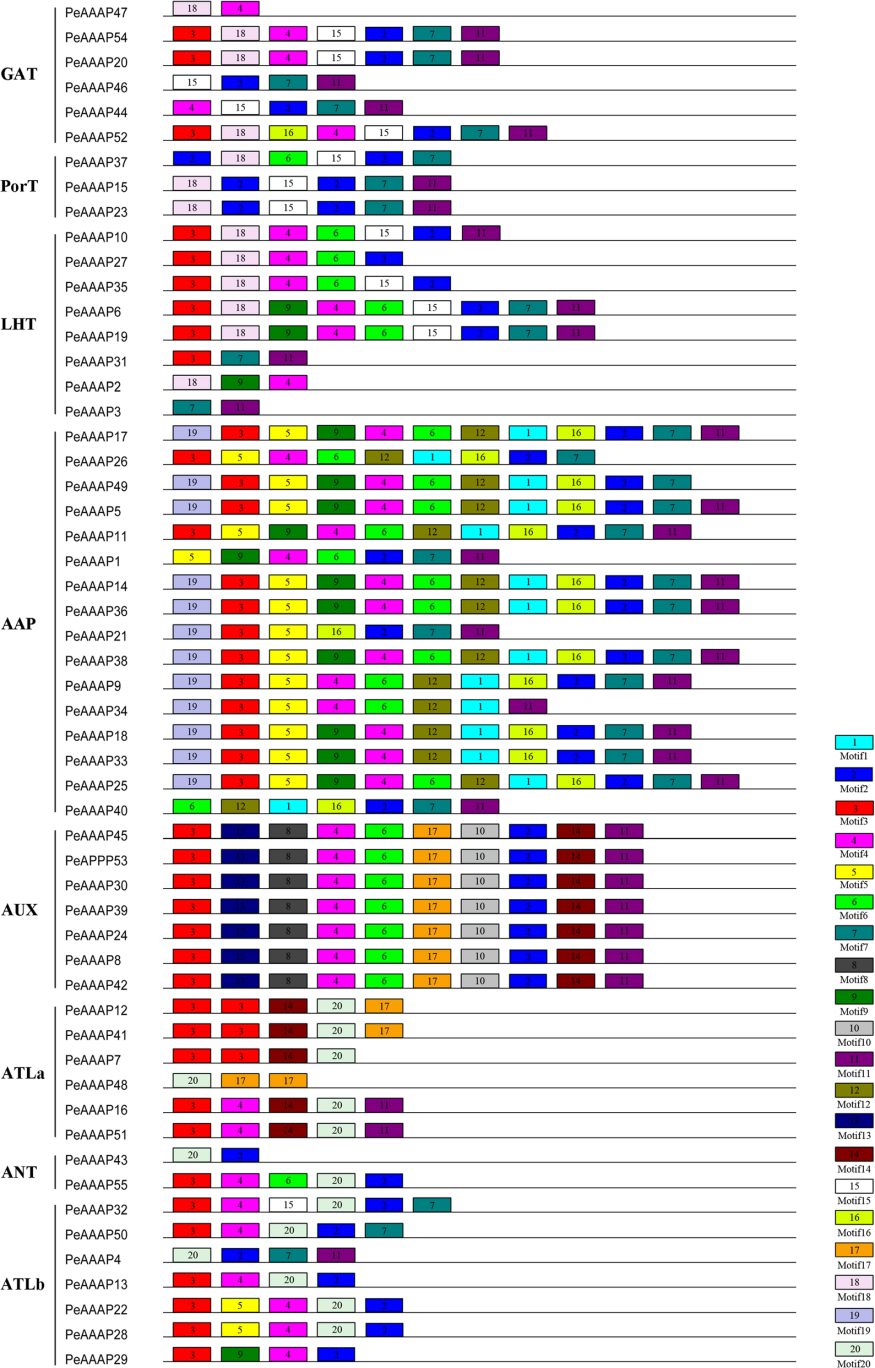
Schematic representation of the 20 conserved motifs in PeAAAP proteins. Conserved motifs of the PeAAAP proteins were identified using the online MEME program based on 55 full length amino acid sequences. Each specific motif was marked by different colored box, and their names in the center of the boxes. The length of each box in the figure didn’t represent the actual motif size

The phylogengtics tree was constructed based on the alignment of full-length amino acid sequences to evaluate the evolutionary relationship among these members of AAAP family. The genes were divided into eight distinct subfamilies, and the AAP subfamily is the largest one of all. To further examine the diversification of the *PeAAAP* genes, 20 distinct motifs (Fig. 2) were found using MEME web server (http://meme-suite.org/tools/meme). Detailed information about the 20 putative motifs included names, widths and best possible matches were listed in Additional file 2: Table S1. The functions of each motif were identified by searching Pfam and SMART database, showing that nine motifs (1 - 6, 9, 10 and 15) encode Aa-trans domains, while the remaining 11 motifs do not encode any domain. Interestingly, some motifs were specific to only one or two subfamilies. For example, motifs 8, 10 and 13 are exclusively found in the AUX subfamily, and motif 1 is only present in the AAP subfamily. Motifs 2 and 4 are found in all of the subfamilies except the ATLa and PorT subfamily, respectively. All members of the AUX subfamily have similar numbers of motifs (Fig. 2), indicating that the structures of members are highly conserved in the same subfamily.

### Evolutionary patterns and divergence of the AAAP gene family in moso bamboo, rice and maize

**Fig. 3:**
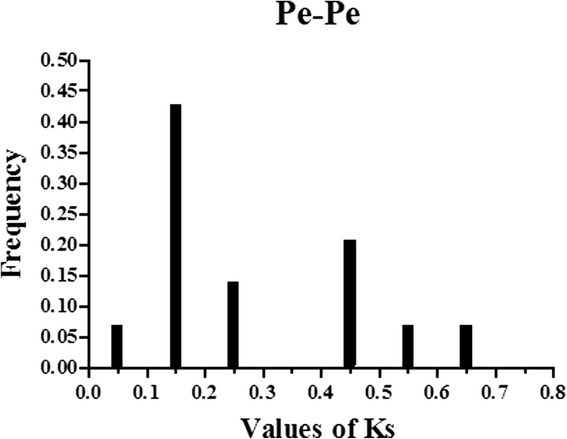
Ks value distribution of *AAAP* genes in the genome of moso bamboo viewed through the frequency distribution of relative Ks modes. Distribution of Ks values were obtained from paralogous gene-pairs (*Pe-Pe*) in the moso bamboo genome

**Fig. 4:**
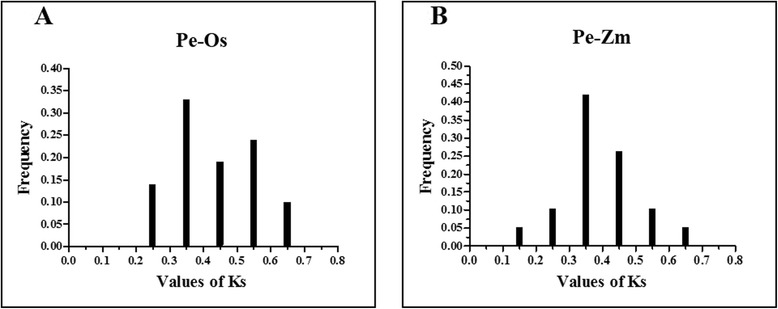
Ks value distribution of *AAAP* genes in the genomes of moso bamboo, rice and maize, viewed through the frequency distribution of relative Ks modes. Distribution of Ks values were obtained from orthologous gene-pairs between moso bamboo and rice (**a**), and, between moso bamboo and maize (**b**)

We identified 14 paralogs (Pe-Pe) in the moso bamboo genome and 21 orthologs (Pe-Os) between moso bamboo and rice, and 19 orthologs (Pe-Zm) between moso bamboo and maize, using phylogeny-based and bidirectional best-hit methods. The formula T = Ks/2λ was used to evaluate the divergence times between moso bamboo, maize and rice, and relative Ks values were used as a proxy for time. All of the paralogous and orthologous pairs are listed in Table 2. The distribution of Ks values of paralogous pairs (Pe-Pe) peaked at approximately 0.15, as shown in Fig. 3. This result suggests that AAAP genes in moso bamboo have undergone a large-scale duplication event approximately 12 million years ago (MYA). The Ks values distribution of orthologous genes (Pe-Os and Pe-Zm) is shown in Fig. 4a and Fig. 4b, respectively. Interestingly, both Pe-Os and Pe-Zm had the same peak at approximately 0.35, showing that these genes have diverged approximately 27 MYA. A previous study showed that bamboo underwent whole-genome duplication 7–12 MYA, while rice and moso bamboo, as well as maize and moso bamboo, diverged 48.6 and 64.6 MYA, respectively [29]. It indicated that the AAAP family has undergone gene evolution after its separation from rice and maize. In general, Ka/Ks ratio less than 1, equal to 1 and greater than 1 means negative or stabilizing selection, neutral selection and positive selection, respectively [46, 47]. To investigate the trend of Ka/Ks ratio in the coding sequences of each gene pairs, we performed sliding-window analysis of Pe-Pe (Additional file 3: Figure S2)*,* Pe-Os (Additional file 4: Figure S3) and Pe-Zm (Additional file 5: Figure S4) gene segments, indicating that the AAAP domains have undergone strong purifying selection (Ka/Ks < < 1) during the process of evolution.

**Table 2:** Paralogous (Pe*-*Pe) and orthologous (Pe-Os and Pe-Zm) gene pairs

Pe-Pe	Pe-Zm	Pe-Os
*PeAAAP45/PeAAAP53*	*PeAAAP43/ZmAAAP70*	*PeAAAP17/OsAAP4*
*PeAAAP13/PeAAAP22*	*PeAAAP30/ZmAAAP1*	*PeAAAP38/OsAAP8*
*PeAAAP14/PeAAAP36*	*PeAAAP38/ZmAAAP21*	*PeAAAP35/OsLHT5*
*PeAAAP18/PeAAAP33*	*PeAAAP1/ZmAAAP59*	*PeAAAP12/OsATL6*
*PeAAAP44/PeAAAP52*	*PeAAAP34/ZmAAAP29*	*PeAAAP46/OsGAT1*
*PeAAAP16/PeAAAP51*	*PeAAAP26/ZmAAAP6*	*PeAAAP55/OsANT2*
*PeAAAP10/PeAAAP27*	*PeAAAP20/ZmAAAP25*	*PeAAAP52/OsGAT3*
*PeAAAP5/PeAAAP11*	*PeAAAP35/ZmAAAP8*	*PeAAAP50/OsATL13*
*PeAAAP25/PeAAAP40*	*PeAAAP44/ZmAAAP23*	*PeAAAP6/OsLHT1*
*PeAAAP28/PeAAAP29*	*PeAAAP55/ZmAAAP53*	*PeAAAP26/OsAAP14*
*PeAAAP43/PeAAAP55*	*PeAAAP41/ZmAAAP62*	*PeAAAP41/OsATL5*
*PeAAAP21/PeAAAP38*	*PeAAAP49/ZmAAAP9*	*PeAAAP7/OsATL4*
*PeAAAP12/PeAAAP41*	*PeAAAP24/ZmAAAP32*	*PeAAAP37/OsProT1*
*PeAAAP26/PeAAAP49*	*PeAAAP32/ZmAAAP27*	*PeAAAP32/OsATL15*
	*PeAAAP46/ZmAAAP19*	*PeAAAP24/OsAUX5*
	*PeAAAP7/ZmAAAP58*	*PeAAAP34/OsAAP19*
	*PeAAAP37/ZmAAAP20*	*PeAAAP4/OsATL14*
	*PeAAAP50/ZmAAAP11*	*PeAAAP28/OsATL9*
	*PeAAAP16/ZmAAAP61*	*PeAAAP9/OsAAP9*
		*PeAAAP1/OsAAP18*
		*PeAAAP21/OsAAP7*

### Putative promoter region analysis

*Cis*-elements play critical roles in plant growth and development, including determining the tissue-specific or stress-responsive expression patterns of genes, and multi-stimulus-responsive genes are closely correlated with *cis*-regulatory elements in their promoter regions [48, 49]. *Cis*-elements have decisive effects on binding to target genes. In this study, we identified three type *cis*-elements, including cold-responsive, drought-responsive and salt-responsive elements in the promoter regions to help elucidate the potential functions of *AAAP* genes in moso bamboo [50, 51]. Numerous *cis*-elements were widespread in the promoter regions, such as S000176 and S000415 for drought stress, S000453 for salt stress and S000407 for cold stress (Additional file 6: Table S2). Moreover, contrasting with the *cis*-regulatory elements of salt stress, there was the higher amount of *cis*-elements for drought and cold stress. These results suggest that transcription factors that regulate *AAP* genes may respond to abiotic stress and have the potential for improving abiotic stress responses, especially drought and cold. These findings may be helpful for further investigating stress tolerance mechanisms in moso bamboo.

### Comparative analysis of AAAP genes in moso bamboo, rice and maize

**Fig. 5:**
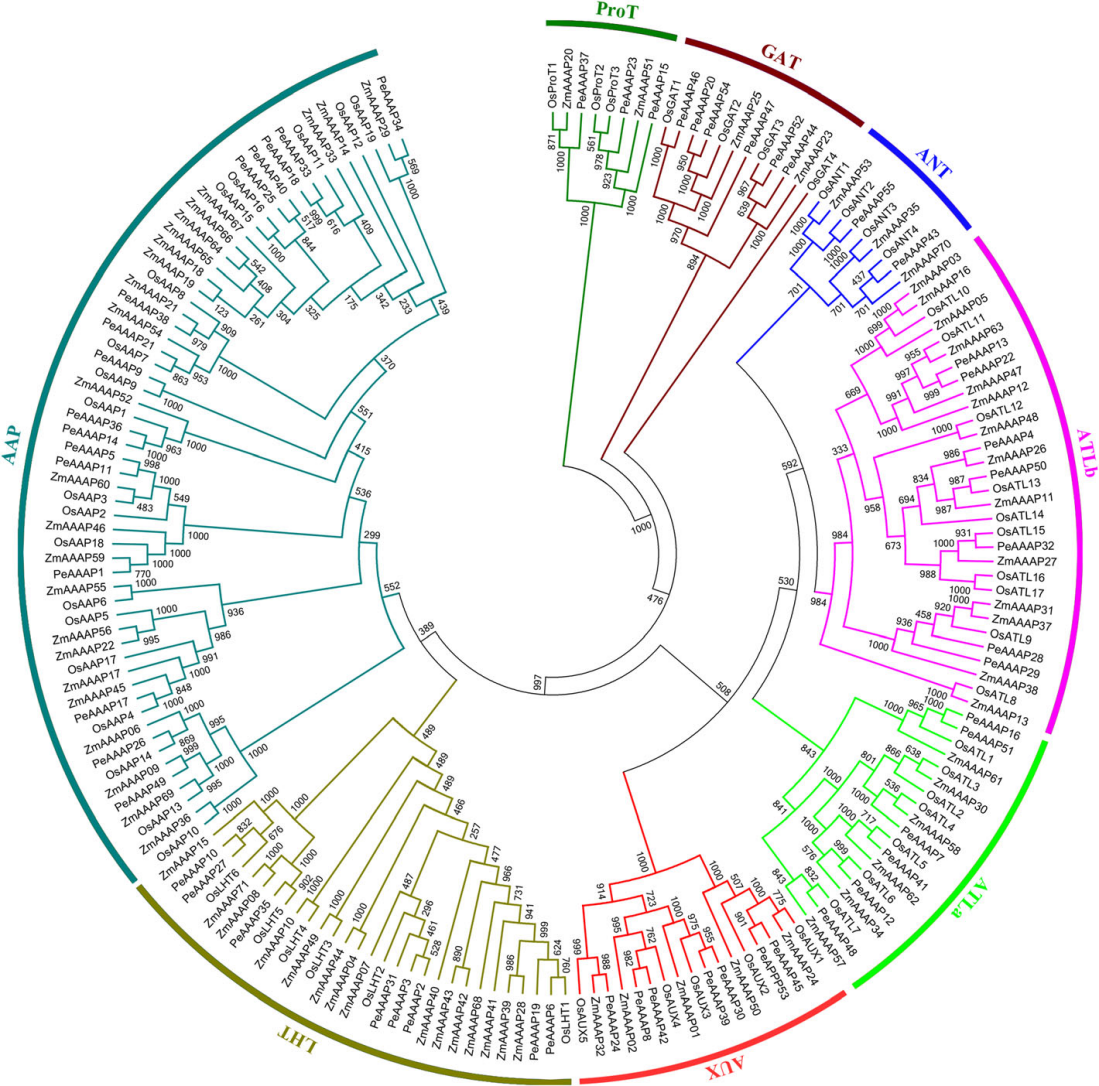
Phylogeny of AAAP proteins from moso bamboo, rice and maize. The tree was generated with Clustal X 2.0 software using the neighbour-joining (N-J) method

**Fig. 6:**
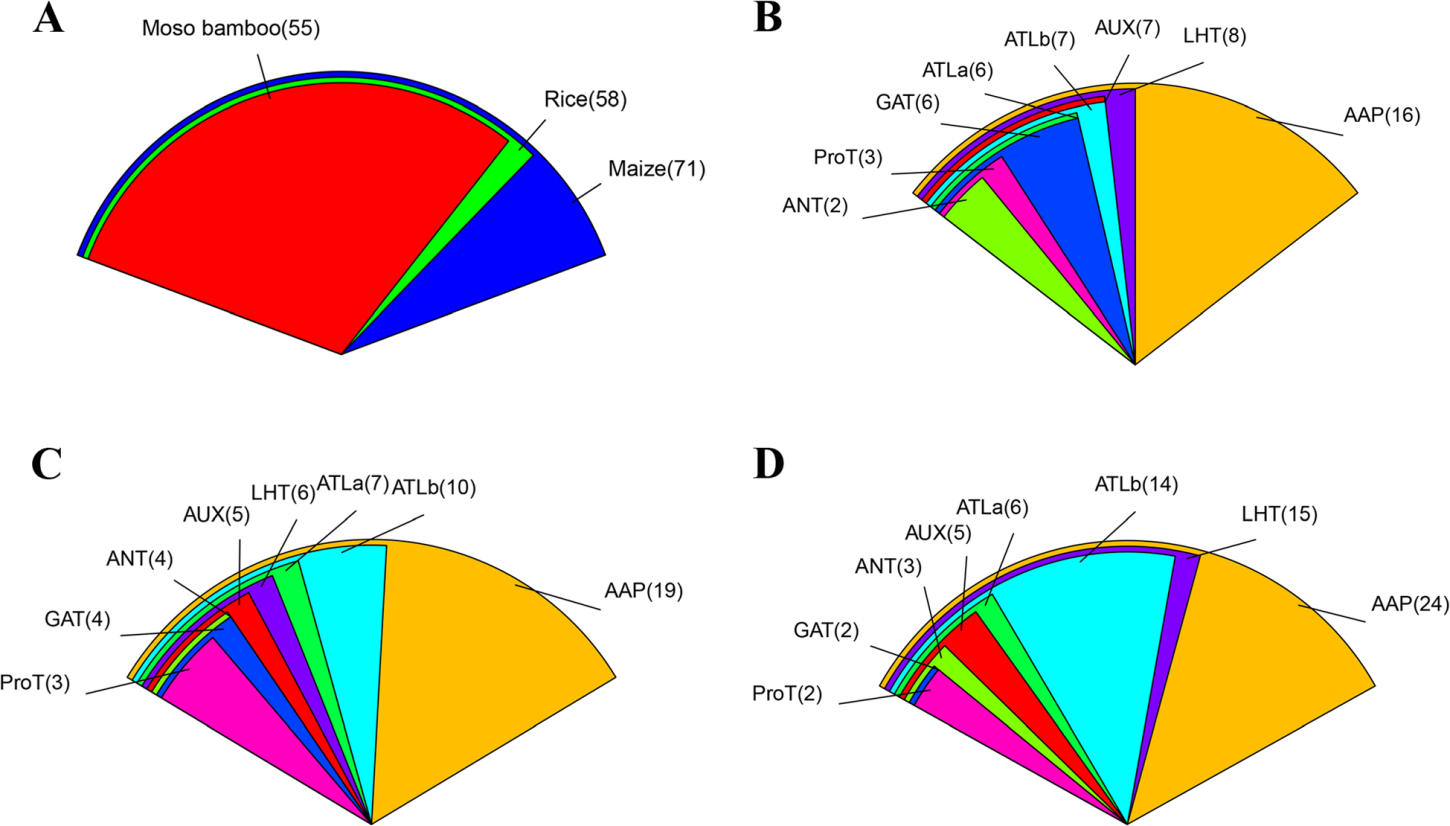
Comparison of AAAP proteins from moso bamboo, rice and maize. **a**: Comparison of AAAP proteins from moso bamboo, rice and maize; different color represents different species, and numbers of AAAP proteins in each species were marked. **b**, **c** and **d** showed that distribution and comparison of each subfamily for moso bamboo, rice and maize, respectively; different subfamilies were represented with different colors, and numbers of members in each subfamily were marked

To date, most studies have focused on analyzing the AAAP family in rice and maize. To further analyze the evolutionary relationships between *AAAP* genes in moso bamboo, rice and maize, we constructed an N-J phylogenetic tree (Fig. 5) of AAAPs using ClustalX 2.11 based on 184 full-length AAAP protein sequences, including 55 sequences from moso bamboo, 58 from rice and 71 from maize. The detailed characteristics of the AAAP genes from rice and maize are listed in Additional file 7: Table S3. The phylogenetic tree clearly shows that the 184 AAAP proteins could be divided into eight distinct groups. A count of the number of AAAP proteins in every moso bamboo (Fig. 6b), rice (Fig. 6c) and maize (Fig. 6d) subfamily was performed. The result is consistent with the previous reports that AAP subfamily is the largest one in AAAP family among these three species. We also found that moso bamboo contains fewer *AAAP* genes than rice and maize (Fig. 6a), suggesting that the *PeAAAP* genes have undergone a gene loss event after divergence from the last common ancestor of moso bamboo, rice and maize.

### Differential expression profiling of moso bamboo AAAP genes

In general, the overall analysis of gene expression profiles in different tissues will contribute to study the dynamic gene expression of *AAAP* genes in moso bamboo. The high-throughput RNA sequencing (RNA-Seq), as one of essential next generation sequencing technology, will allow to reveal a snapshot of RNA presence and quantity from a genome at a given moment in time [52, 53]. In addition, the draft genome sequence of moso bamboo has been released [29]. By now, many studies of expression profiles in several gene families were reported and mainly focused on different tissues [54–56]. While, the genome-wide expression profile of *PeAAAP* genes still remains unclear.

**Fig. 7:**
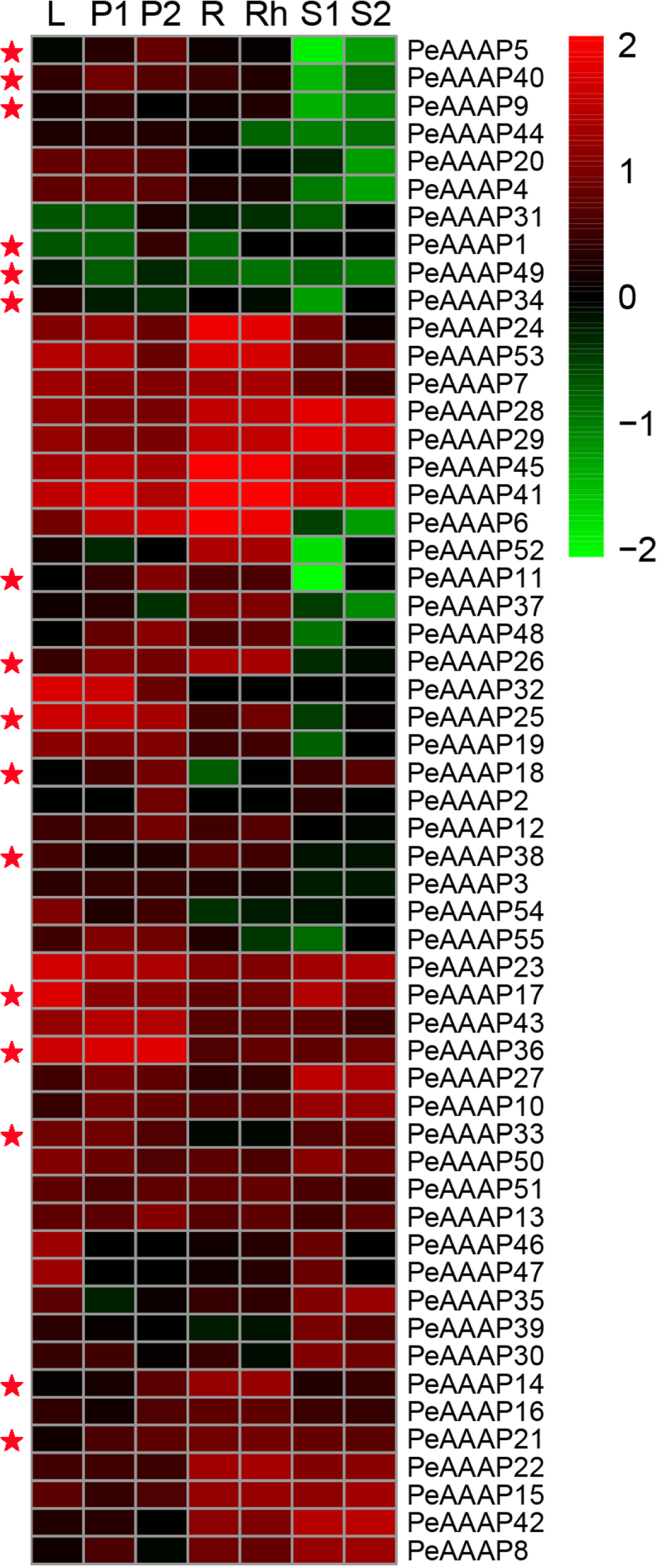
Expression profiles of moso bamboo AAAP genes across different tissues and development stages. Heatmap showing hierarchical clustering of 55 *PeAAAP* genes across different tissues analyzed. Color scale erected vertically at the right side of the picture represents log10 expression values, green represents low level and red indicates high level of transcript abundances. L, leaf; P1, early panicle; P2, advanced panicle; R, root; Rh, rhizome; S1, 20-cm shoot; S2, 50-cm shoot. Every member of AAP subfamily was marked by red stars

We performed a microarray analysis to determine the expression level of each *AAAP* genes on different tissues in moso bamboo based on the above advantages. Finally, the heatmap was produced (Fig. 7) based on the microarray data of 55 moso bamboo *AAAP* (Additional file 8: Table S4) genes downloaded from the NCBI. From the microarray results, it was apparent that a few *AAAP* genes exhibited tissue-specific expression patterns. For instance, two genes (*PeAAAP1* and *PeAAAP5*) and *PeAAAP34* were highly expressed in advanced panicle and leaves, respectively. While the rest members of *PeAAAP* genes showed express at least two tissues. Above all, there are twenty-one genes (*PeAAAP7, PeAAAP10, PeAAAP13, PeAAAP14, PeAAAP15, PeAAAP16, PeAAAP17, PeAAAP27, PeAAAP28, PeAAAP29, PeAAAP36, PeAAAP41, PeAAAP43, PeAAAP45, PeAAAP50, PeAAAP51 and PeAAAP53*) that were widely expressed in all these seven tissues or developmental stages, implying their essential roles in the process of moso bamboo growth and development.

Six gene pairs (*PeAAAP12/PeAAAP41, PeAAAP14/PeAAAP36, PeAAAP21/PeAAAP38, PeAAAP26/PeAAAP49, PeAAAP43/PeAAAP55 and PeAAAP44/PeAAAP52*) of these above identified exhibit distinct expression patterns in different tissues or developmental stages, suggesting that duplicated genes may have different evolutionary fates. For instance, *PeAAAP43* is expressed at a high level in rhizome and shoots, however, its counterpart *PeAAAP55* shows slight relative expression level. By contrast, remaining gene pairs have the same or similar patterns of expression accumulation.

### qRT-PCR analysis of moso bamboo AAAP genes

The phylogenetic analysis indicated that the AAP subfamily contains 16 *PeAAAP* genes and that these genes are closely related to stress-responsive genes in rice. This observation prompted us to investigate possible stress-responsive genes among the 16 *PeAAAP* genes by qRT-PCR. We investigated the expression levels of 16 selected AAP subfamily members (*PeAAAP1*, *PeAAAP5*, *PeAAAP9*, *PeAAAP11*, *PeAAAP14*, *PeAAAP17*, *PeAAAP18*, *PeAAAP21*, *PeAAAP25*, *PeAAAP26*, *PeAAAP33*, *PeAAAP34*, *PeAAAP36*, *PeAAAP38*, *PeAAAP40* and *PeAAAP49*) in the leaves of young seedlings in response to PEG, salt and cold treatment using qRT-PCR. The specific primers used in qRT-PCR analysis of these genes are shown in Additional file 9: Table S5.

**Fig. 8:**
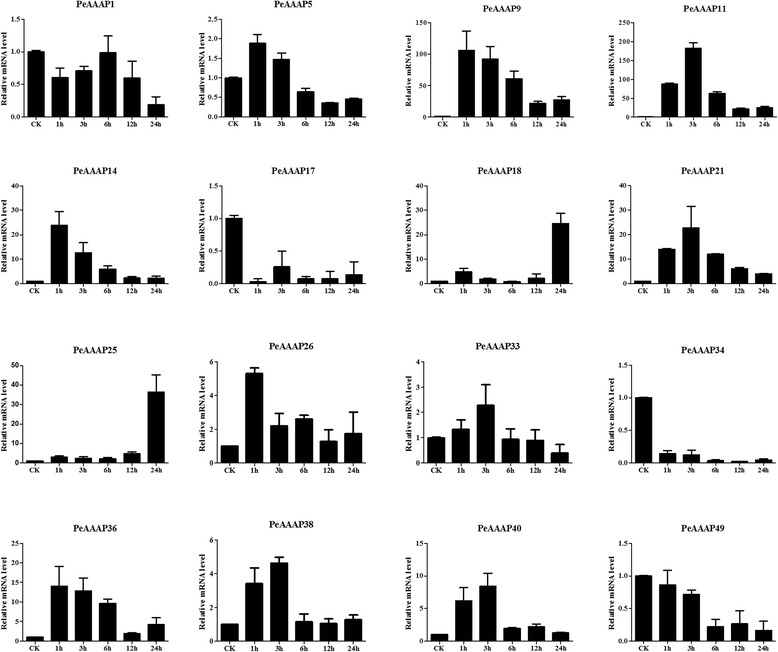
Expression patterns of 16 selected AAP subfamily genes in moso bamboo under PEG treatment, as revealed by qRT-PCR. The Y-axis and X-axis indicates relative expression levels and the time courses of stress treatments, respectively. Error bars, 6 ± SE

For the PEG (drought) treatment (Fig. 8), 10 of the 16 genes (*PeAAAP5*, *PeAAAP9*, *PeAAAP11*, *PeAAAP14*, *PeAAAP18*, *PeAAAP21*, *PeAAAP25*, *PeAAAP2*6, *PeAAAP33, PeAAAP36*, *PeAAAP38* and *PeAAAP40*) were distinctly upregulated in response to PEG (drought) treatment. Four genes (*PeAAAP9*, *PeAAAP14*, *PeAAAP26* and *PeAAAP36*) were the most highly expressed during early (1 h) treatment, followed by a decrease in expression during subsequent treatment (Fig. 8). The expression of two genes (*PeAAAP18* and *PeAAAP25*) peaked at 24 h. *PeAAAP11*, *PeAAAP21*, *PeAAAP38* and *PeAAAP40* were upregulated under 3 h PEG (drought) stress treatment and downregulated at later time points, especially *PeAAAP11*, with an expression level over 150-fold higher at 3 h than at 0 h. By contrast, three genes (*PeAAAP17*, *PeAAAP34* and *PeAAAP40*) were downregulated under PEG (drought) stress treatment (Fig. 8). In addition, there are three genes (*PeAAAP1*, *PeAAAP5* and *PeAAAP33*) were specific, showing slight (<4-fold that at 0 h) changes of expression level in response to PEG (drought) treatment.

**Fig. 9:**
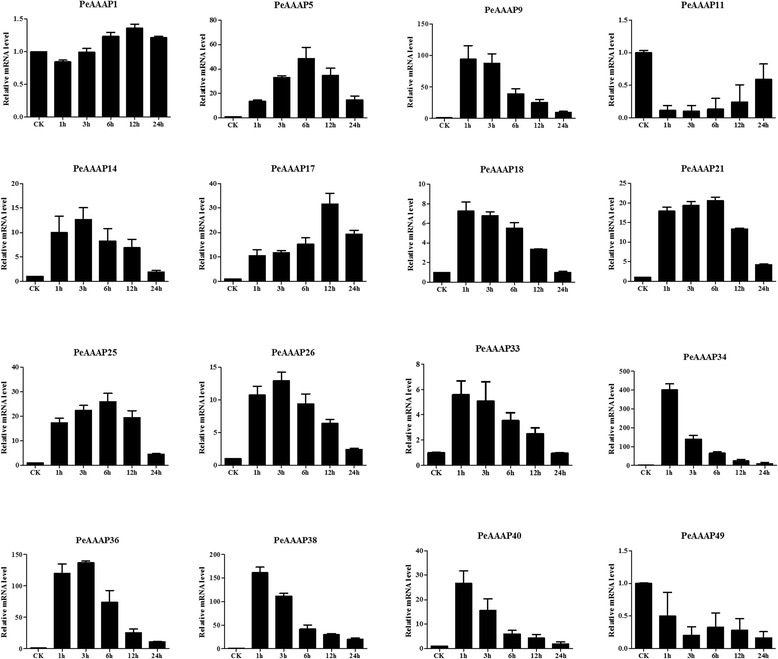
Expression patterns of 16 selected AAP subfamily genes in moso bamboo under cold treatment, as revealed by qRT-PCR. The Y-axis and X-axis indicates relative expression levels and the time courses of stress treatments, respectively. Error bars, 6 ± SE

Two genes in the AAP subfamily (*PeAAAP11* and *PeAAAP49*) were downregulated by cold stress, whereas 13 other genes of this subfamily (*PeAAAP5*, *PeAAAP9*, *PeAAAP14*, *PeAAAP17*, *PeAAAP18*, *PeAAAP21*, *PeAAAP25*, *PeAAAP26*, *PeAAAP33*, *PeAAAP34*, *PeAAAP36*, *PeAAAP38* and *PeAAAP40*) were clearly upregulated by this treatment. Six genes (*PeAAAP9*, *PeAAAP18*, *PeAAAP33*, *PeAAAP34*, *PeAAAP38* and *PeAAAP40*) were the most highly expressed during early (1 h) treatment, and their expression gradually decreased at all later time points (Fig. 9). Moreover, these genes were dramatically upregulated at the 1 h time point, especially *PeAAAP9*, *PeAAAP34* and *PeAAAP38* (more than 150-fold that of control levels; Fig. 9). The expression of *PeAAAP1* changed only slightly over the 24-h time course. Three genes (*PeAAAP5, PeAAAP21* and *PeAAAP25*) exhibited similar trends in expression, with a gradual increase in expression during the early time points, a peak at 6 h and a significant, gradual decrease at all later time points. The expression of *PeAAAP14*, *PeAAAP26* and *PeAAAP36* peaked at 3 h, while the expression level of *PeAAAP17* was highest at 12 h (Fig. 9).

**Fig. 10:**
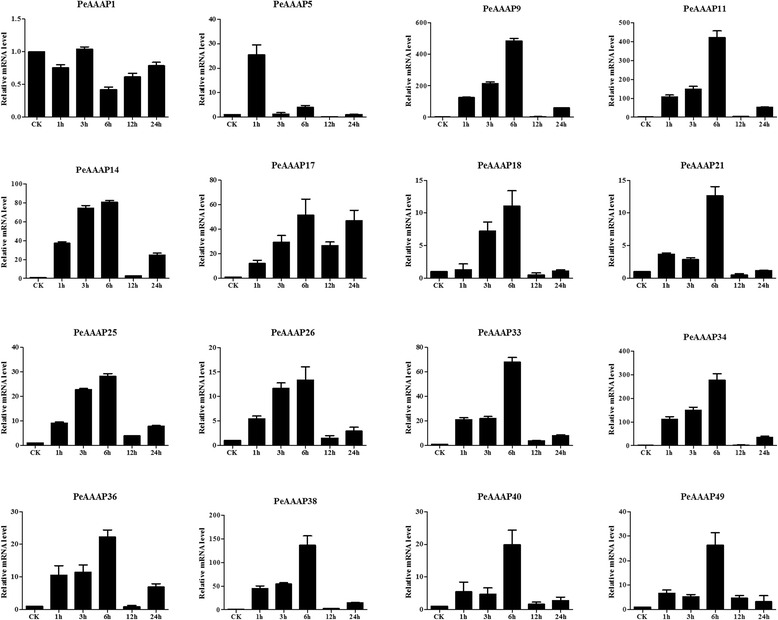
Expression patterns of 16 selected AAP subfamily genes in moso bamboo under NaCl treatment, as revealed by qRT-PCR. The Y-axis and X-axis indicates relative expression levels and the time courses of stress treatments, respectively. Error bars, 6 ± SE

All 16 genes in the AAP subfamily (*PeAAAP1*, *PeAAAP5*, *PeAAAP9*, *PeAAAP11*, *PeAAAP14*, *PeAAAP17*, *PeAAAP18*, *PeAAAP21*, *PeAAAP25*, *PeAAAP26*, *PeAAAP33*, *PeAAAP34*, *PeAAAP36*, *PeAAAP38*, *PeAAAP40* and *PeAAAP49*) were upregulated under NaCl (salt) stress treatment except for *PeAAAP1* (Fig. 10). Only one gene (*PeAAAP5*) showed the highest expression level at 1 h, whereas the 14 other genes (*PeAAAP9*, *PeAAAP11*, *PeAAAP14*, *PeAAAP17*, *PeAAAP18*, *PeAAAP21*, *PeAAAP25*, *PeAAAP26*, *PeAAAP33*, *PeAAAP34*, *PeAAAP36*, *PeAAAP38*, *PeAAAP40* and *PeAAAP49*) exhibited similar expression patterns, with gradual increases in expression at the early time points and significant, gradual decreases at all later time points; the expression of all 14 genes peaked at 6 h (Fig. 10).

In summary, the qRT-PCR results revel that 13 genes *(PeAAAP9*, *PeAAAP11*, *PeAAAP14*, *PeAAAP17*, *PeAAAP18*, *PeAAAP21*, *PeAAAP25*, *PeAAAP26*, *PeAAAP34, PeAAAP36*, *PeAAAP38*, *PeAAAP40,* and *PeAAAP49*) exhibited significant changes in response to all three stress treatments, showing that these genes may exhibit different responses to abiotic stress. It was not difficult to found that these results accord with the putative promoter analysis results of AAP subfamily members (Additional file 6: Table S2). There were several *cis*-elements showing a widely distribution in these members, such as elements S000415 (ACGT), S000407 (CANNTG) and S000453 (GAAAAA) in drought, cold and salt stress response, respectively. These results suggested that a number of *PeAAAP* genes might play crucial roles in regulating abiotic stress responses.

## Discussion

As an indispensable gene family, the eukaryotic-specific AAAP family plays a pivotal role in the process of plant growth and development, which is one of the largest families of AATs identified to date [21, 22]. According to previous studies, several *AAAP* genes have been characterized both physiologically and genetically, such as Arabidopsis AAP and AUX subfamily [18, 25, 57, 58], and rice AAP subfamily [27, 28]. By contrast, AAAP family members have not previously been characterized in moso bamboo. Therefore, in the current study, we identified and characterized 55 predicted *AAAP* genes in moso bamboo using genome wide analysis, and compared these with 58 *OsAAAPs* and 71 *ZmAAAPs*, showing that the number of AAAP genes in moso bamboo (55) is the fewest among these three species [22, 23]. The 184 AAAP proteins were found to be divided into eight distinct subfamilies, with every subfamily containing different members from these three species, meaning that AAAP genes had diversified before moso bamboo, rice and maize split. A count of these subfamilies in different species shows that AAP subfamily is the largest one. And *AAAP* genes with the similar structure showed a tendency to be grouped into the same subfamily, we sought some more valuable evidence to support the reliability of the subfamily classification, including gene structure, motif compositions and TM regions. Furthermore, all members in AUX subfamily have the same numbers and types of TM regions, exon/intron structures and motif compositions, suggesting that these members share a closer evolutionary relationship in the process of AAAP evolution. These results are in accordance with the results of a previous study of the AAAP family in poplar [21]. Furthermore, these results may show that the functions diversified among different members of *AAAP* genes in mso bamboo. In addition, a previous study showed that *AAP1* had 11 TMs in Arabidopsis, which was different from AAP subfamily in moso bamboo, signifying the divergence between different species.

Recent gene duplication events, which help organisms adapt to different environments during growth and development [59, 60] as well as are an important evolutionary mechanism for the rapid expansion and evolution of gene families [46]. To better explain the patterns of macroevolution in moso bamboo, we calculated the value of Ks and Ka in moso bamboo, maize and rice. Specifically, we estimated the Ks and Ka models of paralogous genes (Pe-Pe) and orthologous genes (Pe-Os and Pe-Zm) and calculated the Ks value for each gene pair. We estimated that a large-scale duplication event was occurred approximately 12 MYA in moso bamboo and that the divergence times for orthologous genes (Pe-Os and Pe-Zm) was approximately 27 MYA. Peng *et al.* estimated that the divergence time between moso bamboo and rice was 48.6 MYA, and maize was 64.6 MYA [29]. Ratio of nonsynonymous to synonymous substitutions (Ka/Ks) can be used to measure the history of selection acting on coding sequences [61]. In general, Ka/Ks ratio less than 1, equal to 1 and greater than 1 means negative or stabilizing selection, neutral selection and positive selection, respectively [46, 47]. Interestingly, in this study, the Ka/Ks ratios were less than 1, evidencing that the homologous gene pairs of AAAP family in moso bamboo have undergone a markedly purifying selection in the course of evolution.

The overall analysis of gene expression profiles in different tissues will contribute to study the dynamic gene expression of *AAAP* genes in moso bamboo. Therefore, we displayed the gene expression profiles of 55 identified *PeAAAP* genes using published transcriptome data in NCBI database. Among them, twenty-one genes (*PeAAAP7, PeAAAP10, PeAAAP13, PeAAAP14, PeAAAP15, PeAAAP16, PeAAAP17, PeAAAP27, PeAAAP28, PeAAAP29, PeAAAP36, PeAAAP41, PeAAAP43, PeAAAP45, PeAAAP50, PeAAAP51* and *PeAAAP53*) exhibited relatively high expression level in all seven different tissues or developmental stages, suggesting their importance in the processes of moso bamboo growth and development. While a few numbers of *AAAP* genes show tissue-specific in this analysis.

In plant, many stress-related genes generated a series of stress responses to meet the adverse environmental condition during growth and development. AAAPs are highly regulated by environmental signals and play positive roles in abiotic stress responses in many plants [62, 63]. *AAP* genes were previously studied in several species, leading to the identification of 18 and 8 genes in the AAP subfamily in rice and Arabidopsis, respectively. The expression pattern of a gene can provide significant clues about its function, as demonstrated by Zhao *et al.* [64]. Thus, we performed qRT-PCR to investigate the expression patterns of *PeAAAP* genes under PEG, cold and NaCl treatment. The PeAAP subfamily members showed significantly differential expression patterns under the three abiotic stresses examined. Most *PeAAP* genes were upregulated by all three abiotic stress treatments, suggesting that *PeAAP* genes may play crucial roles in abiotic stress responses in moso bamboo. For instance, *PeAAAP9* was highly expressed (over 100-fold that of control levels) under PEG (drought), cold and salt stress treatment. However, this gene shows a relatively lower expression frequency in leaf, meaning that *PeAAAP9* responds to environmental stress. And conversely, some genes are unresponsive, especially *PeAAAP1* has low expression level in both microarray and qRT-PCR analysis. Furthermore, there were six paralogous pairs in AAP subfamily. Of these gene pairs, five of them (*PeAAAP14/PeAAAP36*, *PeAAAP18/PeAAAP33*, *PeAAAP21/PeAAAP38*, *PeAAAP25/PeAAAP40* and *PeAAAP26/PeAAAP49*) under cold treatment and two gene pairs (*PeAAAP14/PeAAAP36* and *PeAAAP18/PeAAAP33*) under salt treatment (NaCl) had similar expression levels and tendency in the same paralogous pair. These results might suggest that homologous genes had similar putative functions in processes of organism growth and development.

## Conclusions

In this study, we investigated phylogenetic, gene structure, promoter region, divergence time, microarray analysis and qRT-PCR analysis of the 55 predicted AAAP genes in moso bamboo. The qRT-PCR was used to explore the expression patterns of 16 selected AAAP genes under three abiotic stresses: drought (20% PEG-6000), salt (200 mM NaCl) and cold (cultured at 4 °C). These results of this study increase our understanding of AAAP family members, including their possible contributions to abiotic stress responses and other putative functions in process of moso bamboo growth and development.

## Additional files

Additional file 1: Figure S1. Prediction of the transmembrane regions of 55 PeAAAPs. The transmembrane regions of the 55 *PeAAAPs* were predicted using the TMHMM Server v2.0 (http://www.cbs.dtu.dkservicesTMHMM). (TIF 606 kb)
Additional file 2: Table S1. MEME motif sequences and lengths of *AAAP* gene family proteins in moso bamboo. (DOC 38 kb)
Additional file 3: Figure S2. Sliding-window analysis of Pe-Pe. (TIF 92 kb)
Additional file 4: Figure S3. Sliding-window analysis of Pe-Os. (TIF 130 kb)
Additional file 5: Figure S4. Sliding-window analysis of Pe-Zm. (TIF 121 kb)
Additional file 6: Table S2. Summary of abiotic-stress inducible *cis*-elements in the promoter regions of AAP subfamily genes in moso bamboo. The names of 16 AAP genes (*PeAAAP1, -5, -9, -11, -14, -17, -18, -21, -25, -26, -33, -34, -36, 38, -40, -49*) were represented by *Pe1, -5, -9, -11, -14, -17, -18, -21, -25, -26, -33, -34, -36, 38, -40, -49*, respectively. (DOCX 17 kb)
Additional file 7: Table S3. Detailed information about *AAAP* genes in rice and maize. CDS, coding sequence; bp, base pair; aa, amino acids; MW, molecular weight; pI, isoelectric point; Da, Dalton. (DOCX 23 kb)
Additional file 8: Table S4. Microarray data of 55 *AAAP* genes in moso bamboo. These primary data was downloaded from NCBI, and then the relative expression level (log10 expression values) of 7 different issues or development stages was obtained after a series of manual processing. L, leaf; P1, early panicle; P2, advanced panicle; R, root; Rh, rhizome; S1, 20-cm shoot; S2, 50-cm shoot. (DOCX 18 kb)
Additional file 9: Table S5. Primers used for qRT-PCR of 16 selected genes. (DOCX 14 kb)

## Abbreviations

AAAP: Amino acid/auxin permease
AAP: Amino acid permease
AAT: Amino acid transporter
ANT: Aromatic and neutral amino acid transporter
ATL: Amino acid transporter-like
AUX: Auxin transporter
CDS: Coding sequence
GABA: Gamma amino acid butyric acid
GAT: GABA transporter
GDB: Genome database
GSDS: Gene structures display server
LHT: Lysine histidine transporter
MYA: Million years ago
NCBI: National center of biotechnology information
NCGR: National center for gene research
N-J: Neighbour-Joining
PEG: Polyethylene glycol
ProT: Proline transporter
qRT-PCR: Quantitative real-time PCR
RNA-seq: RNA sequencing
SMART: Simple modular architecture research tool
SRA: Short read archive
TIP: Tonoplast intrinsic protein
TMHMM: Transmembrane hidden markov model.
