# Supplementary figures and images for: Genome-Wide analysis of the AAAP gene family in moso bamboo (Phyllostachys edulis)
Source: BMC Plant Biol. 2017 Jan 31;17:29. doi: 10.1186/s12870-017-0980-z (PMC5282885; doi:10.1186/s12870-017-0980-z)

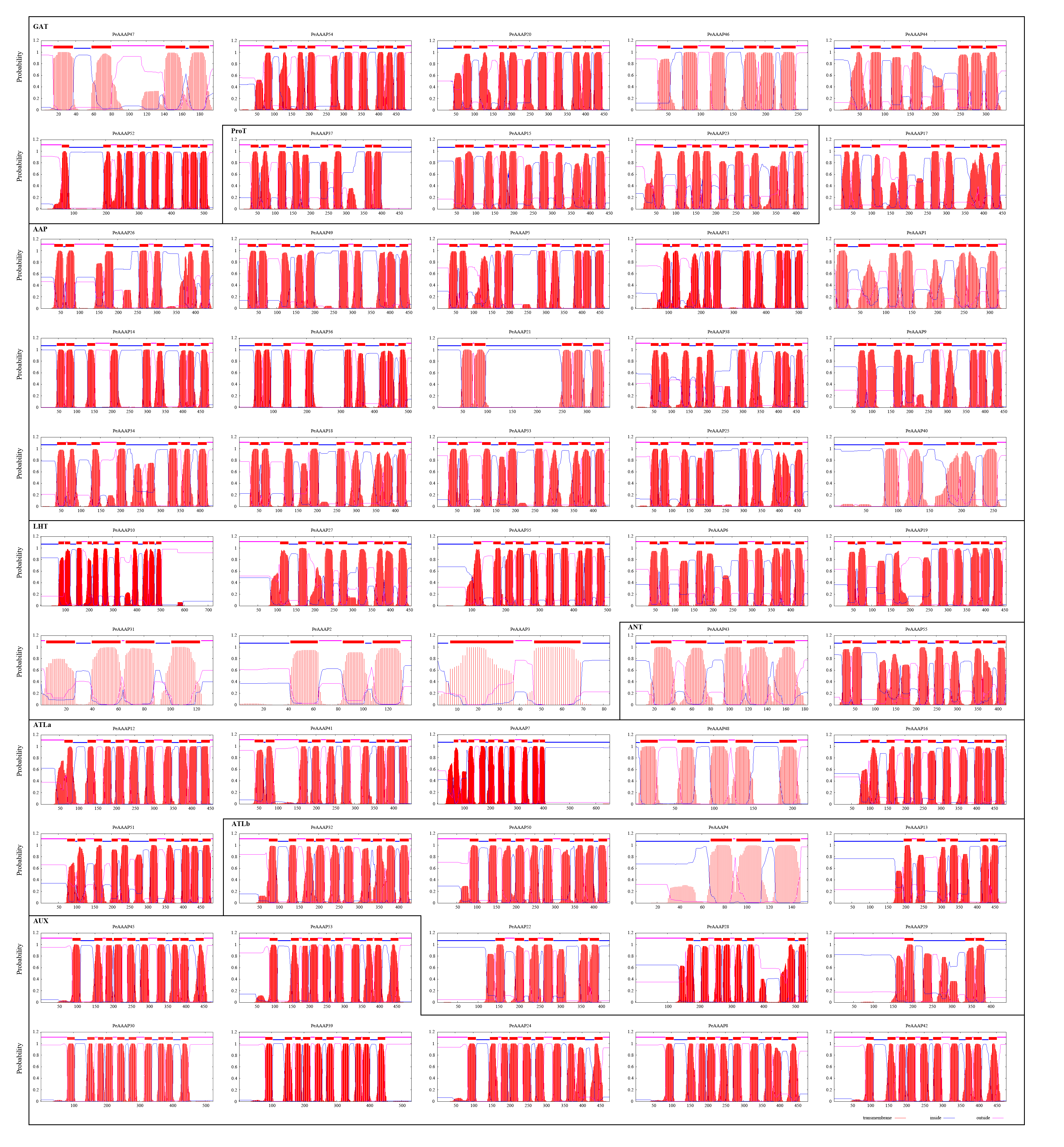

Supplement: Additional file 1: Figure S1. — Prediction of the transmembrane regions of 55 PeAAAPs. The transmembrane regions of the 55 PeAAAPs were predicted using the TMHMM Server v2.0 (http://www.cbs.dtu.dkservicesTMHMM). (TIF 606 kb) [file 12870_2017_980_MOESM1_ESM.tif]

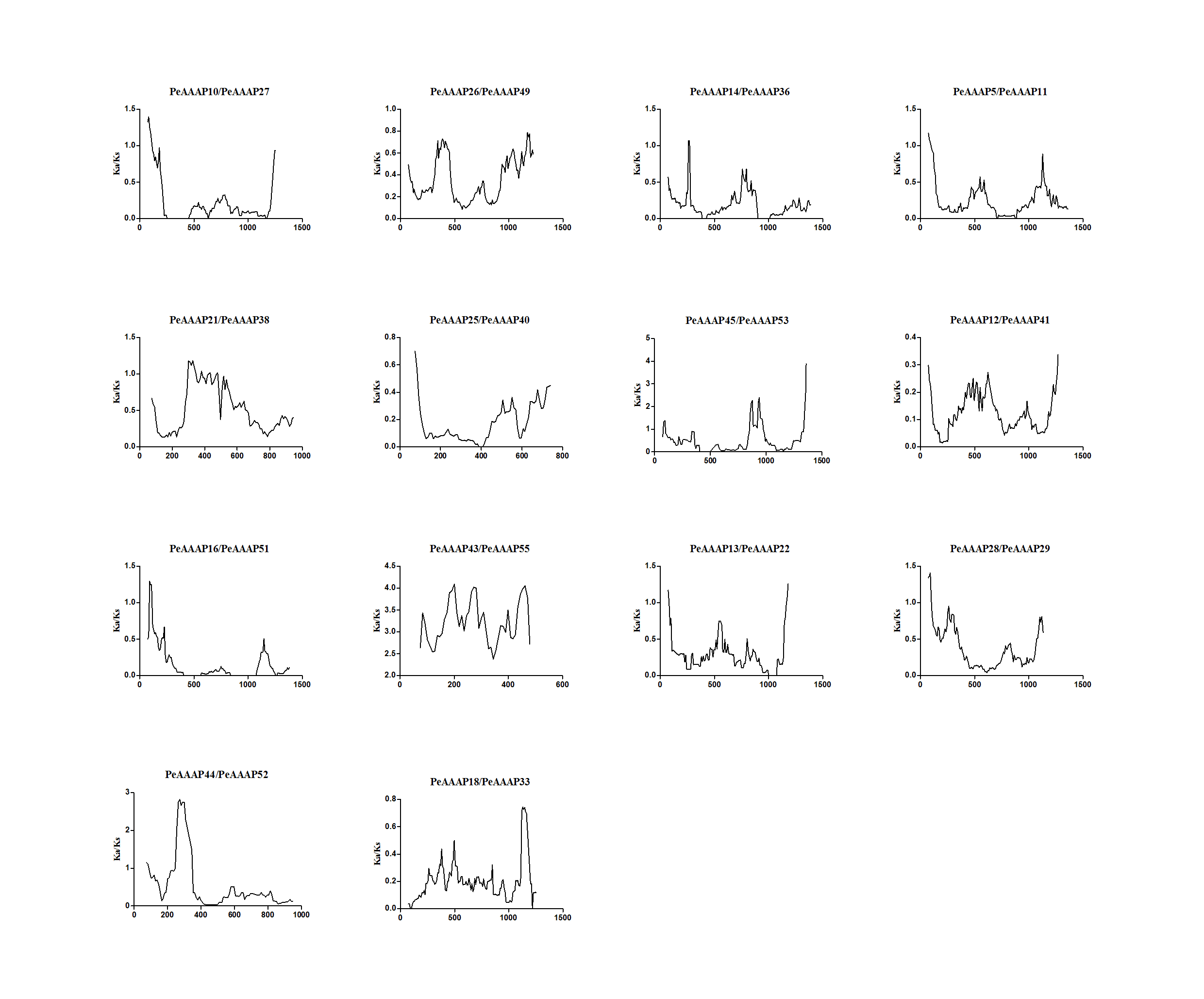

Supplement: Additional file 3: Figure S2. — Sliding-window analysis of Pe-Pe. (TIF 92 kb) [file 12870_2017_980_MOESM3_ESM.tif]

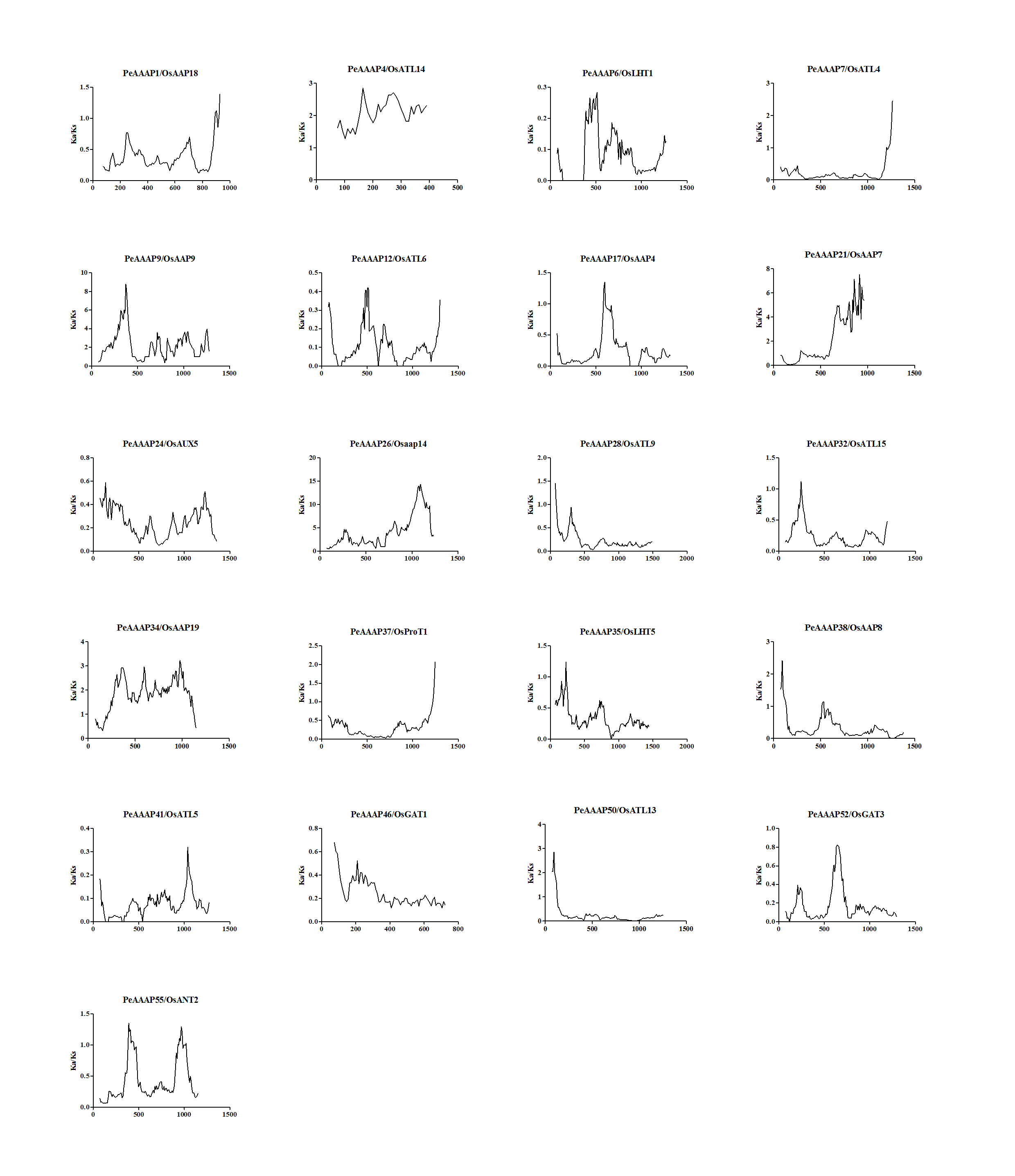

Supplement: Additional file 4: Figure S3. — Sliding-window analysis of Pe-Os. (TIF 130 kb) [file 12870_2017_980_MOESM4_ESM.tif]

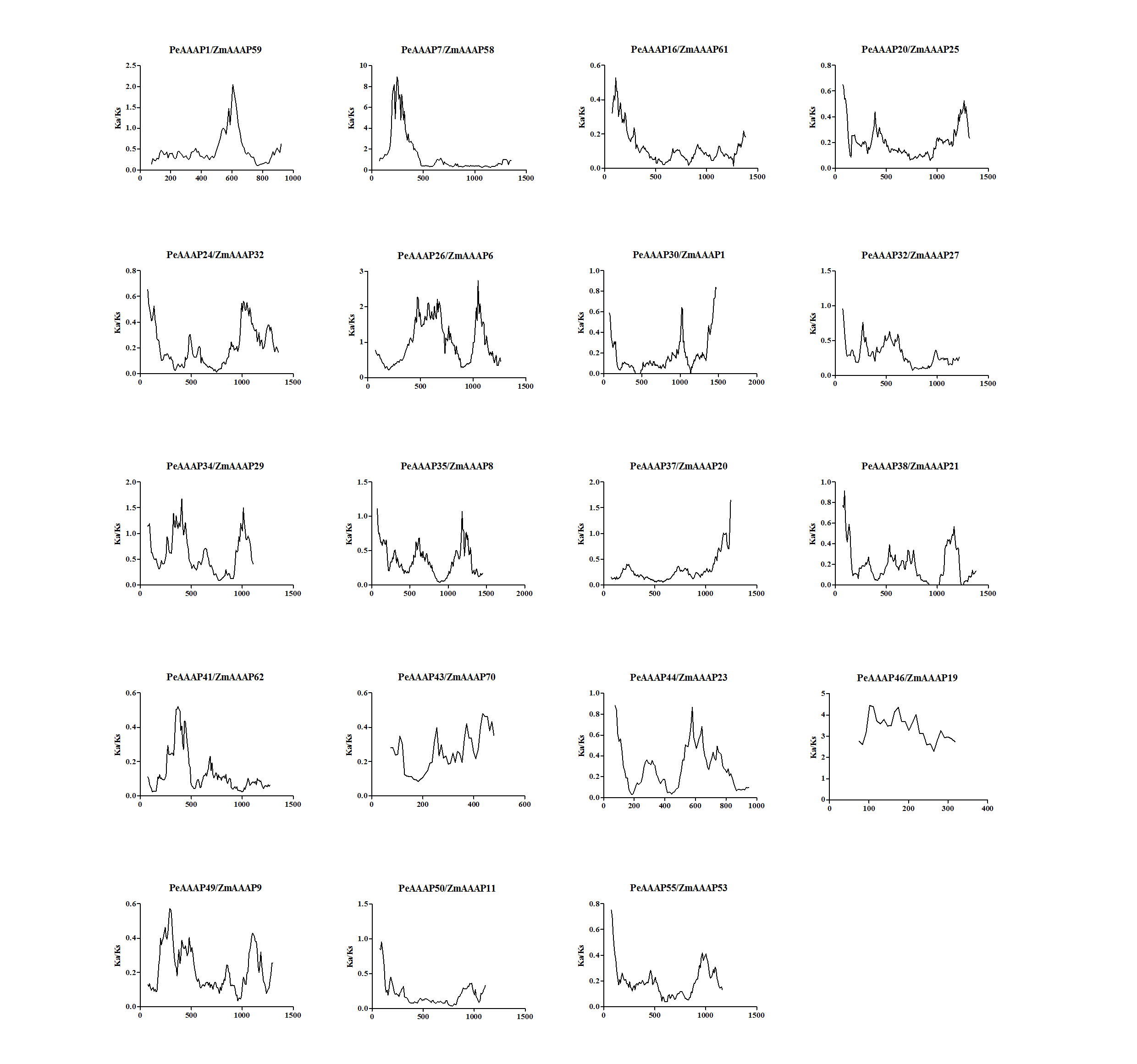

Supplement: Additional file 5: Figure S4. — Sliding-window analysis of Pe-Zm. (TIF 121 kb) [file 12870_2017_980_MOESM5_ESM.tif]
