# Supplementary material for: Genome-Wide analysis of the AAAP gene family in moso bamboo (Phyllostachys edulis)
Source: BMC Plant Biol. 2017 Jan 31;17:29. doi: 10.1186/s12870-017-0980-z (PMC5282885; doi:10.1186/s12870-017-0980-z)
Supplement: Additional file 2: Table S1. — MEME motif sequences and lengths of AAAP gene family proteins in moso bamboo. (DOC 38 kb) [file 12870_2017_980_MOESM2_ESM.doc]

Table S1. The MEME motif sequences and lengths of AAAP gene family proteins in moso bamboo.

| Motif | Width | Best possible match |
| --- | --- | --- |
| 1 | 50 | CGCMGYAAFGDDAPDNLLTGFGFYEPFWLIDIANVCIVVHLIGAYQVFCQ |
| 2 | 27 | RTAYVCFTTFIAMMFPFFGDIMGLVGA |
| 3 | 29 | WFHCITNQIGQGVLSLPWSIKQLGWVWGP |
| 4 | 29 | IFGVCQIVFSQIPNFHNIWWWSFVAAVMS |
| 5 | 41 | MLCFSFVTYYTSTLLADCYRSGDPVTGKRNYTYMDAVRSNL |
| 6 | 35 | VDHTPPQKIWRYFQALGDIAFAYSYHNVTIEIQHT |
| 7 | 29 | FWPLTVYFPCEMYICQKKPPRWSTRWCCN |
| 8 | 50 | NHVIQWFEVLDGLLGRHWRNVGLAFNCTFLLFGSVIQLIACASNIYYIND |
| 9 | 41 | CGVIQYVNLFGVAIGYTITASISMRAIKRANCFHKNGHKND |
| 10 | 50 | WAFGDELLTHSNAFALLPRTGWRDAAVVLMLIHQFITFGFACTPLYFVWE |
| 11 | 21 | AAAWGSIRNIIDDLKTYKPFK |
| 12 | 21 | PPPPENKTMKKATMISVSTTT |
| 13 | 29 | QLFYGLMGSWTAYLISILYVEYRTRKERE |
| 14 | 35 | SFTVYIIPAMAHMCTFRSQQARENAVEKPPRFMGK |
| 15 | 41 | IQATLKPPVHKKMWKGLCLCYTVVAFCFYPVAITGYWAYGN |
| 16 | 29 | PIFQFVEKWAAARWPDSEFINREHEVKPP |
| 17 | 29 | WRPQKFKYIYLMATLYVLTLTLPSASAMY |
| 18 | 23 | HEEVPGKRHDRYRELAQHVFGEK |
| 19 | 15 | DDDGRPKRTGTMWTA |
| 20 | 29 | TAIMGYLMFGEDTESQVTLNFDENKGISK |
