# Supplementary material for: Genome-Wide analysis of the AAAP gene family in moso bamboo (Phyllostachys edulis)
Source: BMC Plant Biol. 2017 Jan 31;17:29. doi: 10.1186/s12870-017-0980-z (PMC5282885; doi:10.1186/s12870-017-0980-z)
Supplement: Additional file 6: Table S2. — Summary of abiotic-stress inducible cis-elements in the promoter regions of AAP subfamily genes in moso bamboo. The names of 16 AAP genes (PeAAAP1, -5, -9, -11, -14, -17, -18, -21, -25, -26, -33, -34, -36, 38, -40, -49) were represented by Pe1, -5, -9, -11, -14, -17, -18, -21, -25, -26, -33, -34, -36, 38, -40, -49, respectively. (DOCX 17 kb) [file 12870_2017_980_MOESM6_ESM.docx]

Table S2. Summary of abiotic-stress inducible *cis*-elements is in the promoter regions of AAP subfamily genes in moso bamboo. Cis-elements with larger numbers were marked red.

| Abiotic stress | Cis-element | motif sequence | Pe1 | Pe5 | Pe9 | Pe11 | Pe14 | Pe17 | Pe18 | Pe21 | Pe25 | Pe26 | Pe33 | Pe34 | Pe36 | Pe38 | Pe40 | Pe49 |
| --- | --- | --- | --- | --- | --- | --- | --- | --- | --- | --- | --- | --- | --- | --- | --- | --- | --- | --- |
| Drought-stress | S000133 | CCACGTGG | 0 | 0 | 0 | 0 | 0 | 0 | 0 | 0 | 0 | 0 | 0 | 0 | 0 | 0 | 0 | 0 |
|  | S000153 | CCGAC | 0 | 1 | 3 | 5 | 1 | 0 | 2 | 2 | 3 | 6 | 3 | 3 | 0 | 2 | 0 | 1 |
|  | S000174 | CACATG | 2 | 0 | 4 | 4 | 1 | 1 | 2 | 2 | 2 | 0 | 3 | 1 | 1 | 1 | 5 | 1 |
|  | S000175 | CTAACCA | 0 | 0 | 0 | 0 | 0 | 0 | 0 | 2 | 0 | 0 | 0 | 0 | 0 | 0 | 1 | 1 |
|  | S000176 | CNGTTR | 8 | 3 | 3 | 4 | 2 | 6 | 10 | 4 | 6 | 9 | 2 | 3 | 3 | 7 | 7 | 8 |
|  | S000177 | TAACTG | 1 | 0 | 0 | 1 | 0 | 0 | 1 | 0 | 0 | 2 | 0 | 0 | 0 | 0 | 0 | 0 |
|  | S000402 | ACCGAC | 0 | 0 | 1 | 0 | 0 | 0 | 0 | 1 | 0 | 2 | 0 | 0 | 0 | 0 | 0 | 0 |
|  | S000408 | WAACCA | 0 | 1 | 2 | 2 | 1 | 0 | 1 | 4 | 2 | 3 | 0 | 2 | 1 | 3 | 4 | 3 |
|  | S000413 | CATGTG | 2 | 0 | 4 | 4 | 1 | 1 | 2 | 2 | 2 | 0 | 3 | 1 | 1 | 0 | 5 | 1 |
|  | S000414 | ACGTG | 1 | 3 | 2 | 3 | 2 | 3 | 3 | 2 | 4 | 3 | 4 | 0 | 0 | 1 | 3 | 3 |
|  | S000415 | ACGT | 6 | 18 | 16 | 10 | 6 | 12 | 10 | 8 | 14 | 14 | 10 | 2 | 6 | 6 | 8 | 12 |
|  | S000418 | RCCGAC | 0 | 0 | 1 | 1 | 0 | 0 | 1 | 1 | 3 | 3 | 1 | 0 | 0 | 1 | 0 | 0 |
|  | total |  | 20 | 26 | 36 | 34 | 14 | 23 | 32 | 28 | 36 | 42 | 26 | 12 | 12 | 21 | 33 | 30 |
| Cold-stress | S000153 | CCGAC | 0 | 1 | 3 | 5 | 1 | 0 | 2 | 2 | 3 | 6 | 3 | 3 | 0 | 2 | 0 | 1 |
|  | S000402 | ACCGAC | 0 | 0 | 1 | 0 | 0 | 0 | 0 | 1 | 0 | 2 | 0 | 0 | 0 | 0 | 0 | 0 |
|  | S000407 | CANNTG | 10 | 14 | 18 | 26 | 8 | 14 | 20 | 16 | 24 | 16 | 26 | 18 | 18 | 12 | 26 | 22 |
|  | S000418 | RCCGAC | 0 | 0 | 1 | 1 | 0 | 0 | 1 | 1 | 3 | 3 | 1 | 0 | 0 | 1 | 0 | 0 |
|  | total |  | 10 | 15 | 23 | 32 | 9 | 14 | 23 | 20 | 30 | 27 | 30 | 21 | 18 | 15 | 26 | 23 |
| Salt-stress | S000402 | ACCGAC | 0 | 0 | 1 | 0 | 0 | 0 | 0 | 1 | 0 | 2 | 0 | 0 | 0 | 0 | 0 | 0 |
|  | S000418 | RCCGAC | 0 | 0 | 1 | 1 | 0 | 0 | 1 | 1 | 3 | 3 | 1 | 0 | 0 | 1 | 0 | 0 |
|  | S000453 | GAAAAA | 0 | 3 | 5 | 5 | 1 | 5 | 3 | 3 | 5 | 4 | 8 | 3 | 2 | 4 | 3 | 3 |
|  | total |  | 0 | 3 | 7 | 6 | 1 | 5 | 4 | 5 | 8 | 9 | 9 | 3 | 2 | 5 | 3 | 3 |
