# Supplementary material for: Genome-Wide analysis of the AAAP gene family in moso bamboo (Phyllostachys edulis)
Source: BMC Plant Biol. 2017 Jan 31;17:29. doi: 10.1186/s12870-017-0980-z (PMC5282885; doi:10.1186/s12870-017-0980-z)
Supplement: Additional file 7: Table S3. — Detailed information about AAAP genes in rice and maize. CDS, coding sequence; bp, base pair; aa, amino acids; MW, molecular weight; pI, isoelectric point; Da, Dalton. (DOCX 23 kb) [file 12870_2017_980_MOESM7_ESM.docx]

Table S3. Detailed information about AAAP genes in rice and maize.

| **Gene name** | **Sequences ID** | **Mol.Wt.(KDa)** | **PI** | **Length of CDS** | **Size(aa)** |
| --- | --- | --- | --- | --- | --- |
| OsAAP1 | LOC_Os07g04180 | 52.86 | 8.66 | 1464 | 487 |
| OsAAP2 | LOC_ Os06g12330 | 51.9 | 8.32 | 1455 | 484 |
| OsAAP3 | LOC_Os06g36180 | 52.78 | 7.93 | 1464 | 487 |
| OsAAP4 | LOC_Os12g09300 | 50.89 | 8.27 | 1407 | 468 |
| OsAAP5 | LOC_Os01g65660 | 50 | 8.77 | 1398 | 465 |
| OsAAP6 | LOC_Os01g65670 | 50.09 | 8.68 | 1401 | 466 |
| OsAAP7 | LOC_Os05g34980 | 53.51 | 8.02 | 1491 | 496 |
| OsAAP8 | LOC_Os01g66010 | 52.87 | 8.28 | 1467 | 488 |
| OsAAP9 | LOC_Os02g01210 | 57.21 | 8.63 | 1557 | 518 |
| OsAAP10 | LOC_Os02g49060 | 50.25 | 8.03 | 1410 | 469 |
| OsAAP11 | LOC_Os11g09020 | 51.35 | 8.77 | 1431 | 476 |
| OsAAP12 | LOC_Os12g09320 | 50.07 | 8.04 | 1407 | 468 |
| OsAAP13 | LOC_Os04g39489 | 50.75 | 8.15 | 1401 | 466 |
| OsAAP14 | LOC_Os04g56470 | 51.38 | 8.64 | 1410 | 469 |
| OsAAP15 | LOC_Os12g08130 | 51.06 | 8.73 | 1428 | 475 |
| OsAAP16 | LOC_Os12g08090 | 51.09 | 8.73 | 1428 | 475 |
| OsAAP17 | LOC_Os06g12350 | 53.44 | 9.55 | 1524 | 507 |
| OsAAP18 | LOC_Os06g36210 | 50.72 | 8.3 | 1425 | 474 |
| OsAAP19 | LOC_Os04g41350 | 43.15 | 8.47 | 1236 | 411 |
| OsLHT1 | LOC_Os08g03350 | 49.83 | 9.45 | 1344 | 447 |
| OsLHT2 | LOC_Os12g14100 | 48.94 | 8.77 | 1341 | 446 |
| OsLHT3 | LOC_Os05g14820 | 49.94 | 9.12 | 1371 | 456 |
| OsLHT4 | LOC_Os04g38860 | 47.8 | 9 | 1335 | 444 |
| OsLHT5 | LOC_Os04g47420 | 55.03 | 9.11 | 1539 | 512 |
| OsLHT6 | LOC_Os12g30040 | 54.95 | 8.97 | 1527 | 508 |
| OsGAT1 | LOC_Os05g50920 | 51.04 | 9.43 | 1446 | 481 |
| OsGAT2 | LOC_Os01g43320 | 48.53 | 9.02 | 1365 | 454 |
| OsGAT3 | LOC_Os01g63854 | 48.77 | 8.73 | 1374 | 457 |
| OsGAT4 | LOC_Os10g27980 | 47.98 | 8.92 | 1329 | 442 |
| OsProT1 | LOC_Os01g68050 | 49.02 | 9.4 | 1344 | 447 |
| OsProT2 | LOC_Os03g44230 | 52.05 | 7.9 | 1422 | 473 |
| OsProT3 | LOC_Os07g01090 | 47.66 | 9.51 | 1305 | 434 |
| OsAUX1 | LOC_Os01g63770 | 54.76 | 8.15 | 1479 | 492 |
| OsAUX2 | LOC_Os05g37470 | 55.66 | 8.65 | 1512 | 503 |
| OsAUX3 | LOC_Os03g14080 | 58.08 | 9.21 | 1575 | 524 |
| OsAUX4 | LOC_Os10g05690 | 59.84 | 8.64 | 1644 | 547 |
| OsAUX5 | LOC_Os11g06820 | 52.96 | 9.33 | 1443 | 480 |
| OsANT1 | LOC_Os07g12770 | 45.71 | 7.01 | 1275 | 424 |
| OsANT2 | LOC_Os03g60260 | 43.57 | 7.79 | 1257 | 418 |
| OsANT3 | LOC_Os02g44980 | 44.85 | 7.53 | 1269 | 422 |
| OsANT4 | LOC_Os04g47780 | 44.79 | 7.84 | 1278 | 425 |
| OsATL1 | LOC_Os06g43700 | 51.76 | 7.55 | 1461 | 486 |
| OsATL2 | LOC_Os09g26290 | 32.74 | 8.61 | 927 | 308 |
| OsATL3 | LOC_Os02g49510 | 48.11 | 7.18 | 1347 | 448 |
| OsATL4 | LOC_Os06g16420 | 48.12 | 7.3 | 1347 | 448 |
| OsATL5 | LOC_Os06g42720 | 49.98 | 6.42 | 1377 | 458 |
| OsATL6 | LOC_Os02g09810 | 50.15 | 6.45 | 1380 | 459 |
| OsATL7 | LOC_Os01g61044 | 47.87 | 9.8 | 1380 | 459 |
| OsATL8 | LOC_Os11g19240 | 50.23 | 9.08 | 1452 | 483 |
| OsATL9 | LOC_Os02g54730 | 5.93 | 5.07 | 1647 | 548 |
| OsATL10 | LOC_Os12g38570 | 63.41 | 4.63 | 1785 | 594 |
| OsATL11 | LOC_Os02g01100 | 62.91 | 5.92 | 1725 | 574 |
| OsATL12 | LOC_Os06g12320 | 43.79 | 9.41 | 1242 | 413 |
| OsATL13 | LOC_Os04g38680 | 47.99 | 8.3 | 1368 | 455 |
| OsATL14 | LOC_Os04g38660 | 24.23 | 9.39 | 690 | 229 |
| OsATL15 | LOC_Os01g41420 | 67.36 | 9.7 | 1896 | 631 |
| OsATL16 | LOC_Os01g41400 | 47.39 | 8.75 | 1332 | 443 |
| OsATL17 | LOC_Os01g40410 | 49.42 | 5.89 | 1383 | 460 |
| OsCAT1 | LOC_Os01g11160 | 65.51 | 8.13 | 1851 | 616 |
| OsCAT2 | LOC_Os02g43860 | 64.44 | 7.57 | 1818 | 605 |
| OsCAT3 | LOC_Os03g43970 | 46.94 | 8.54 | 1329 | 442 |
| OsCAT4 | LOC_Os03g45170 | 68.2 | 5.74 | 1920 | 639 |
| OsCAT5 | LOC_Os04g45950 | 60.78 | 8.3299 | 1686 | 561 |
| OsCAT6 | LOC_Os06g34830 | 61.92 | 8.42 | 1791 | 596 |
| OsCAT7 | LOC_Os10g30090 | 66.05 | 6.73 | 1869 | 622 |
| OsCAT8 | LOC_Os11g05690 | 49.76 | 5.95 | 1413 | 470 |
| OsCAT9 | LOC_Os12g06060 | 56.37 | 7.26 | 1608 | 535 |
| OsCAT10 | LOC_Os12g41890 | 62.6 | 8.44 | 1806 | 601 |
| OsCAT11 | LOC_Os12g42850 | 66.1 | 5.8 | 1866 | 621 |
| OsBAT1 | LOC_Os01g42234 | 57.02 | 8.51 | 1599 | 532 |
| OsBAT2 | LOC_Os01g71700 | 38.64 | 9.055 | 1089 | 521 |
| OsBAT3 | LOC_Os01g71710 | 55.92 | 8.8742 | 1566 | 362 |
| OsBAT4 | LOC_Os01g71720 | 55.99 | 7.76 | 1578 | 525 |
| OsBAT5 | LOC_Os01g71740 | 55.68 | 8.7 | 1554 | 517 |
| OsBAT6 | LOC_Os01g71760 | 54.46 | 9 | 1527 | 508 |
| OsBAT7 | LOC_Os04g35540 | 57.76 | 8.08 | 1593 | 530 |
| OsLAT1 | LOC_Os02g47210 | 60.17 | 6.18 | 1695 | 564 |
| OsLAT2 | LOC_Os03g25840 | 35.29 | 8.98 | 993 | 330 |
| OsLAT3 | LOC_Os03g25869 | 59.97 | 12.42 | 1644 | 547 |
| OsLAT4 | LOC_Os03g25920 | 51.81 | 8.96 | 1500 | 499 |
| OsLAT5 | LOC_Os03g37984 | 60.38 | 7.27 | 1653 | 550 |
| OsLAT6 | LOC_Os08g41370 | 21.29 | 8.97 | 579 | 192 |
| OsLAT7 | LOC_Os12g39080 | 48.99 | 12.27 | 1344 | 447 |
| OsLAT8 | LOC_Os01g19850 | 88.3 | 7.9546 | 2391 | 796 |
| OsLAT9 | LOC_Os08g23440 | 108.45 | 6.2146 | 2970 | 989 |
| ZmAAAP01 | GRMZM2G149481_P01 | 57.98 | 9.14 | 1563 | 520 |
| ZmAAAP02 | GRMZM2G129413_P01 | 62.74 | 9.01 | 1713 | 570 |
| ZmAAAP03 | GRMZM2G017170_P01 | 64.6 | 5.89 | 1794 | 597 |
| ZmAAAP04 | GRMZM2G052461_P01 | 49.49 | 8.59 | 1335 | 444 |
| ZmAAAP05 | GRMZM5G894432_P01 | 31.29 | 5.92 | 891 | 296 |
| ZmAAAP06 | GRMZM2G042933_P01 | 60.28 | 9.17 | 1668 | 555 |
| ZmAAAP07 | GRMZM2G175321_P01 | 48.42 | 9.16 | 1317 | 438 |
| ZmAAAP08 | GRMZM2G109865_P01 | 56.75 | 9.56 | 1584 | 527 |
| ZmAAAP09 | GRMZM2G155491_P01 | 50.37 | 8.38 | 1377 | 458 |
| ZmAAAP10 | GRMZM2G114523_P01 | 43.48 | 9.15 | 1212 | 403 |
| ZmAAAP11 | GRMZM2G137161_P01 | 42.4 | 9.34 | 1212 | 403 |
| ZmAAAP12 | GRMZM2G097802_P03 | 62.02 | 5.87 | 1722 | 573 |
| ZmAAAP13 | GRMZM2G108597_P01 | 49.54 | 8.92 | 1395 | 464 |
| ZmAAAP14 | GRMZM2G082434_P02 | 51.93 | 8.8 | 1440 | 479 |
| ZmAAAP15 | GRMZM2G125832_P01 | 54.88 | 8.97 | 1527 | 508 |
| ZmAAAP16 | GRMZM2G092223_P01 | 61.28 | 5.84 | 1716 | 571 |
| ZmAAAP17 | GRMZM2G032304_P01 | 36.55 | 9.03 | 1008 | 335 |
| ZmAAAP18 | GRMZM2G031167_P01 | 53.52 | 8.84 | 1500 | 499 |
| ZmAAAP19 | GRMZM2G136288_P01 | 13.96 | 4.42 | 396 | 131 |
| ZmAAAP20 | GRMZM2G078024_P01 | 48.83 | 9.27 | 1341 | 446 |
| ZmAAAP21 | GRMZM2G110195_P01 | 53.61 | 8.58 | 1485 | 494 |
| ZmAAAP22 | GRMZM5G830545_P03 | 50.07 | 8.86 | 1407 | 468 |
| ZmAAAP23 | GRMZM2G154958_P01 | 48.86 | 9.08 | 1380 | 459 |
| ZmAAAP24 | GRMZM2G127949_P01 | 54.46 | 8.63 | 1473 | 490 |
| ZmAAAP25 | GRMZM2G057733_P01 | 49.59 | 9.22 | 1404 | 467 |
| ZmAAAP26 | GRMZM2G476954_P01 | 26.81 | 6.4 | 759 | 252 |
| ZmAAAP27 | GRMZM2G066428_P01 | 52.89 | 9.07 | 1497 | 498 |
| ZmAAAP28 | GRMZM2G046743_P01 | 50.59 | 9.06 | 1368 | 455 |
| ZmAAAP29 | GRMZM2G180547_P01 | 51.12 | 8.79 | 1440 | 479 |
| ZmAAAP30 | GRMZM2G427319_P01 | 57.34 | 9.1 | 1599 | 532 |
| ZmAAAP31 | GRMZM2G101125_P01 | 58.72 | 5.42 | 1623 | 540 |
| ZmAAAP32 | GRMZM2G045057_P01 | 53.87 | 8.88 | 1458 | 485 |
| ZmAAAP33 | GRMZM2G332505_P02 | 50.77 | 8.6 | 1419 | 472 |
| ZmAAAP34 | GRMZM2G332562_P01 | 49.08 | 6.19 | 1356 | 451 |
| ZmAAAP35 | GRMZM2G074053_P01 | 45.48 | 6.99 | 1299 | 432 |
| ZmAAAP36 | GRMZM2G161641_P01 | 50.58 | 7.53 | 1422 | 473 |
| ZmAAAP37 | GRMZM2G083788_P01 | 59.02 | 5.08 | 1632 | 543 |
| ZmAAAP38 | GRMZM2G080843_P01 | 57.25 | 4.88 | 1587 | 528 |
| ZmAAAP39 | GRMZM2G180659_P01 | 50.2 | 9.14 | 1359 | 452 |
| ZmAAAP40 | GRMZM2G127342_P01 | 47.83 | 8.66 | 1314 | 437 |
| ZmAAAP41 | GRMZM2G127328_P01 | 51.98 | 8.96 | 1419 | 472 |
| ZmAAAP42 | GRMZM2G127294_P01 | 50.7 | 9.36 | 1365 | 454 |
| ZmAAAP43 | GRMZM2G127338_P01 | 52.31 | 9.04 | 1407 | 468 |
| ZmAAAP44 | GRMZM2G429322_P01 | 54.61 | 9.1 | 1482 | 493 |
| ZmAAAP45 | AC205362.4_FGP002 | 52.12 | 8.48 | 1455 | 484 |
| ZmAAAP46 | GRMZM2G331283_P02 | 50.69 | 5.97 | 1410 | 469 |
| ZmAAAP47 | GRMZM2G134888_P01 | 60.22 | 5.94 | 1668 | 555 |
| ZmAAAP48 | GRMZM2G010433_P01 | 43.24 | 9.2 | 1200 | 399 |
| ZmAAAP49 | GRMZM2G476886_P01 | 49.1 | 9.29 | 1341 | 446 |
| ZmAAAP50 | GRMZM2G067022_P02 | 72.48 | 10.07 | 1956 | 651 |
| ZmAAAP51 | GRMZM5G894233_P02 | 46.93 | 8.9 | 1299 | 432 |
| ZmAAAP52 | GRMZM2G108023_P01 | 52.12 | 8.8 | 1452 | 483 |
| ZmAAAP53 | GRMZM2G173967_P01 | 45.44 | 6.54 | 1287 | 428 |
| ZmAAAP54 | GRMZM2G164814_P01 | 51.98 | 8.51 | 1437 | 478 |
| ZmAAAP55 | GRMZM2G092945_P01 | 49.5 | 8.79 | 1386 | 461 |
| ZmAAAP56 | GRMZM2G096407_P01 | 50.84 | 9.04 | 1455 | 484 |
| ZmAAAP57 | GRMZM2G036448_P01 | 49.23 | 9.54 | 1413 | 470 |
| ZmAAAP58 | GRMZM2G150406_P01 | 51.42 | 5.98 | 1434 | 477 |
| ZmAAAP59 | GRMZM2G145989_P01 | 51.28 | 8.77 | 1416 | 471 |
| ZmAAAP60 | GRMZM2G136300_P01 | 52.95 | 8.9 | 1461 | 486 |
| ZmAAAP61 | GRMZM2G087635_P01 | 51.73 | 6.13 | 1464 | 487 |
| ZmAAAP62 | GRMZM2G177659_P01 | 49.89 | 6.06 | 1380 | 459 |
| ZmAAAP63 | GRMZM2G105192_P05 | 53.74 | 8.69 | 1476 | 491 |
| ZmAAAP64 | GRMZM2G076593_P01 | 51.54 | 8.27 | 1443 | 480 |
| ZmAAAP65 | GRMZM2G433162_P01 | 51.24 | 8.61 | 1434 | 477 |
| ZmAAAP66 | GRMZM2G157168_P01 | 51.67 | 8.46 | 1449 | 482 |
| ZmAAAP67 | GRMZM2G413943_P01 | 140.21 | 5.35 | 3807 | 1268 |
| ZmAAAP68 | GRMZM2G149216_P01 | 49.65 | 9.09 | 1341 | 446 |
| ZmAAAP69 | GRMZM2G173597_P02 | 50.77 | 8.72 | 1407 | 468 |
| ZmAAAP70 | GRMZM2G455128_P01 | 52.4 | 8.5 | 1488 | 495 |
| ZmAAAP71 | GRMZM2G360519_P01 | 55.73 | 9.33 | 1554 | 517 |
