# Supplementary material for: Genome-Wide analysis of the AAAP gene family in moso bamboo (Phyllostachys edulis)
Source: BMC Plant Biol. 2017 Jan 31;17:29. doi: 10.1186/s12870-017-0980-z (PMC5282885; doi:10.1186/s12870-017-0980-z)
Supplement: Additional file 8: Table S4. — Microarray data of 55 AAAP genes in moso bamboo. These primary data was downloaded from NCBI, and then the relative expression level (log10 expression values) of 7 different issues or development stages was obtained after a series of manual processing. L, leaf; P1, early panicle; P2, advanced panicle; R, root; Rh, rhizome; S1, 20-cm shoot; S2, 50-cm shoot. (DOCX 18 kb) [file 12870_2017_980_MOESM8_ESM.docx]

Table S4. The microarray data of 55 AAAP genes in moso bamboo.

| Name | Leaf | Panicle1 | Panicle2 | Root | Rhizome | Shoot20 | Shoot50 |
| --- | --- | --- | --- | --- | --- | --- | --- |
| PeAAAP43 | 1.16421 | 1.48234 | 1.428337 | 0.688878 | 0.717167 | 0.780091 | 0.513916 |
| PeAAAP44 | 0.235448 | 0.316159 | 0.292309 | 0.091034 | -0.80247 | -1.02153 | -0.89706 |
| PeAAAP45 | 1.313766 | 1.52481 | 1.344066 | 2.06992 | 1.947273 | 1.459302 | 1.310037 |
| PeAAAP46 | 1.270171 | 0 | -0.01804 | 0.138612 | 0.323413 | 0.838796 | -0.03567 |
| PeAAAP47 | 1.270171 | 0 | -0.01804 | 0.138612 | 0.323413 | 0.838796 | -0.03567 |
| PeAAAP48 | 0 | 0.800879 | 1.095089 | 0.623221 | 0.770113 | -0.91256 | 0 |
| PeAAAP49 | -0.15362 | -0.72036 | -0.32131 | -0.76762 | -0.94252 | -0.80087 | -1.00822 |
| PeAAAP50 | 1.056421 | 0.83473 | 0.649217 | 0.735348 | 0.615729 | 1.113683 | 0.85116 |
| PeAAAP51 | 0.767712 | 0.61929 | 0.762784 | 0.756521 | 0.821077 | 0.586955 | 0.501209 |
| PeAAAP52 | 0.186818 | -0.30776 | 0 | 1.394585 | 1.33086 | -1.8099 | 0 |
| PeAAAP53 | 1.466488 | 1.405218 | 0.870904 | 1.760402 | 1.730479 | 0.877641 | 1.045542 |
| PeAAAP54 | 1.009472 | 0.252275 | 0.509318 | -0.39848 | -0.21741 | -0.12963 | 0 |
| PeAAAP55 | 0.532361 | 0.998085 | 0.901395 | 0.263695 | -0.42064 | -0.84068 | 0 |
| PeAAAP34 | 0.242482 | -0.21347 | -0.34012 | 0.017926 | -0.08938 | -1.27239 | 0 |
| PeAAAP35 | 0.706791 | -0.252 | 0.117503 | 0.433717 | 0.392211 | 1.042312 | 1.203131 |
| PeAAAP36 | 1.624376 | 1.754697 | 1.793497 | 0.652637 | 0.816972 | 0.779659 | 0.89087 |
| PeAAAP37 | 0.134477 | 0.296577 | -0.37631 | 1.032663 | 1.019536 | -0.4851 | -1.07118 |
| PeAAAP38 | 0.529718 | 0.169818 | 0.292292 | 0.69048 | 0.509815 | -0.12659 | -0.13216 |
| PeAAAP39 | 0.373749 | 0.054916 | 0.003784 | -0.20523 | -0.13342 | 0.988864 | 0.679521 |
| PeAAAP40 | 0.412988 | 0.877171 | 0.700032 | 0.463962 | 0.253963 | -1.47439 | -0.83565 |
| PeAAAP41 | 1.509685 | 1.740051 | 1.427726 | 2.053781 | 2.065232 | 1.770438 | 1.756812 |
| PeAAAP42 | 0.289042 | 0.323887 | -0.03537 | 1.125244 | 1.021069 | 1.489707 | 1.515552 |
| PeAAAP30 | 0.420792 | 0.509897 | 0.060377 | 0.426474 | -0.09301 | 1.05965 | 0.886162 |
| PeAAAP31 | -0.67399 | -0.71012 | 0.245745 | -0.24522 | -0.38384 | -0.70149 | 0 |
| PeAAAP32 | 1.780408 | 1.644551 | 0.869669 | 0 | 0 | 0 | 0 |
| PeAAAP33 | 0.885159 | 0.897691 | 0.631141 | -0.04608 | -0.06124 | 0.640017 | 0.777741 |
| PeAAAP22 | 0.525579 | 0.531386 | 0.486011 | 1.326022 | 1.357331 | 1.044869 | 1.091505 |
| PeAAAP23 | 1.668939 | 1.461414 | 1.40786 | 1.017968 | 1.005631 | 1.319695 | 1.399874 |
| PeAAAP24 | 1.050662 | 1.226262 | 0.865194 | 1.918992 | 1.822866 | 0.948039 | 0.092342 |
| PeAAAP25 | 1.623423 | 1.535376 | 1.362207 | 0.582522 | 0.895568 | -0.46006 | 0.07667 |
| PeAAAP26 | 0.419586 | 1.01808 | 0.949922 | 1.339825 | 1.365033 | -0.33961 | -0.10926 |
| PeAAAP27 | 0.500946 | 0.982772 | 0.764287 | 0.395753 | 0.418914 | 1.502286 | 1.369833 |
| PeAAAP28 | 1.16487 | 1.012753 | 0.962444 | 1.553833 | 1.567552 | 1.836502 | 1.683199 |
| PeAAAP29 | 1.16487 | 1.012753 | 0.962444 | 1.553833 | 1.567552 | 1.836502 | 1.683199 |
| PeAAAP12 | 0.468064 | 0.565557 | 0.921859 | 0.537713 | 0.69007 | 0.025199 | -0.05594 |
| PeAAAP13 | 0.759683 | 0.729861 | 1.062924 | 0.683874 | 0.759199 | 0.534595 | 0.719067 |
| PeAAAP14 | 0.071668 | 0.198 | 0.732995 | 1.198987 | 1.221837 | 0.271144 | 0.417517 |
| PeAAAP15 | 0.72061 | 0.427986 | 0.637374 | 1.171571 | 1.206861 | 1.138877 | 1.289254 |
| PeAAAP16 | 0.391434 | 0.167704 | 0.648732 | 0.781349 | 0.761109 | 0.483862 | 0.430135 |
| PeAAAP17 | 1.758349 | 1.085376 | 1.111659 | 0.777546 | 0.906347 | 1.447896 | 1.051133 |
| PeAAAP18 | 0.002827 | 0.550205 | 0.928118 | -0.70961 | 0 | 0.480106 | 0.68807 |
| PeAAAP19 | 1.118546 | 1.059454 | 1.024875 | 0.480559 | 0.527317 | -0.75319 | 0 |
| PeAAAP20 | 0.806546 | 0.797726 | 0.700575 | 0 | 0 | -0.28564 | -1.23756 |
| PeAAAP21 | 0.137227 | 0.623641 | 0.75951 | 0.883786 | 0.921963 | 0.824218 | 0.74564 |
| PeAAAP5 | -0.07184 | 0.332026 | 0.820248 | 0.094513 | 0.063679 | -1.93402 | -1.27126 |
| PeAAAP8 | 0.141525 | 0.605033 | -0.05622 | 0.897336 | 0.818229 | 1.235796 | 1.24374 |
| PeAAAP6 | 0.943661 | 1.541318 | 1.701614 | 2.018646 | 1.936726 | -0.50315 | -1.24011 |
| PeAAAP10 | 0.426003 | 0.934416 | 0.824041 | 0.690703 | 0.686351 | 1.228998 | 1.16132 |
| PeAAAP3 | 0.375052 | 0.42601 | 0.444828 | 0.289315 | 0.203658 | -0.21066 | -0.19297 |
| PeAAAP4 | 0.769672 | 0.868705 | 0.713627 | 0.239557 | 0.19653 | -0.96919 | -1.27823 |
| PeAAAP7 | 1.273848 | 1.110202 | 1.007432 | 1.262636 | 1.326743 | 0.81621 | 0.524534 |
| PeAAAP9 | 0.131157 | 0.413363 | -0.01845 | 0.147185 | 0.28864 | -1.42164 | -1.07324 |
| PeAAAP1 | -0.68918 | -0.76123 | 0.435261 | -0.79502 | 0 | 0 | 0 |
| PeAAAP11 | -0.00212 | 0.427049 | 1.067881 | 0.611433 | 0.59391 | -2.06173 | 0 |
| PeAAAP2 | 0 | 0 | 0.875326 | 0 | 0 | 0.374002 | 0 |
