# Supplementary material for: Genome-Wide analysis of the AAAP gene family in moso bamboo (Phyllostachys edulis)
Source: BMC Plant Biol. 2017 Jan 31;17:29. doi: 10.1186/s12870-017-0980-z (PMC5282885; doi:10.1186/s12870-017-0980-z)
Supplement: Additional file 9: Table S5. — Primers used for qRT-PCR of 16 selected genes. (DOCX 14 kb) [file 12870_2017_980_MOESM9_ESM.docx]

Table S5. The primers of qRT-PCR of 16 selected genes.

| **Genes** | **Primers used in qRT-PCR(5′→3′)** | |
| --- | --- | --- |
| PeAAAP1 | Forwards | CATCAGTGCCGCTGCTATCA |
|  | Reverse | TCGCAGTCCGCCTTGTG |
| PeAAAP5 | Forwards | GCGCTGGCGCGAACTA |
|  | Reverse | ACTCCAACTTCAGTGCCAGTCA |
| PeAAAP9 | Forwards | CCCGGACCGCGATGT |
|  | Reverse | GTTCTCGTCGATCTTGGAAATGA |
| PeAAAP11 | Forwards | CCCGGACCGCGATGT |
|  | Reverse | GTTCTCGTCGATCTTGGAAATGA |
| PeAAAP14 | Forwards | GCTGTCATGTCCGTGGCTTA |
|  | Reverse | CCCACCTGCTATTGTTGCTATG |
| PeAAAP17 | Forwards | TGCTACCTCGGTCGGAAAAA |
|  | Reverse | GTCCCCCACAGGTTGACGTA |
| PeAAAP18 | Forwards | CTCTTCGCCTTCGTCATCTACTACA |
|  | Reverse | CGCCGGATCGGTAGCA |
| PeAAAP21 | Forwards | CGGAGGCGAAGGTGATGA |
|  | Reverse | ATGCACCCGCACAGCAT |
| PeAAAP25 | Forwards | GGAGGCGAAGGTGATGAAGAG |
|  | Reverse | CCCGCACAGCATGTAGAACA |
| PeAAAP26 | Forwards | CTTTGCGGACCGATGCTT |
|  | Reverse | AACTTGTTCACGAACGCACTGT |
| PeAAAP33 | Forwards | CGGAGGCGAAGGTGATGA |
|  | Reverse | ATGCACCCGCACAGCAT |
| PeAAAP34 | Forwards | TCTCCGTCGTCCTGCTAGAGA |
|  | Reverse | CTAGCAAGATCAAACAGAGCGAGTA |
| PeAAAP36 | Forwards | CCAACTGCTTCCACCATAACG |
|  | Reverse | ATCATGTTTGTTGTGTTGGATGCT |
| PeAAAP38 | Forwards | CATTGCCGCCTCCATCAG |
|  | Reverse | GTCGCTGTGCCCCTTCTG |
| PeAAAP40 | Forwards | CGGAGGCGAATGTGATGAA |
|  | Reverse | ATGCACCCGCACAGCAT |
| PeAAAP49 | Forwards | ATCGCATTCGCTTACCCATACT |
|  | Reverse | GGCTGGTGGCGACTTCAG |
| PeTIP41 | Forwards | AAAATCATTGTAGGCCATTGTCG |
|  | Reverse | ACTAAATTAAGCCAGCGGGAGTG |
